# Li^+^ Dynamics of Liquid Electrolytes Nanoconfined in Metal–Organic Frameworks

**DOI:** 10.1021/acsami.1c16214

**Published:** 2021-11-09

**Authors:** Marco Farina, Benjamin B. Duff, Cristina Tealdi, Andrea Pugliese, Frédéric Blanc, Eliana Quartarone

**Affiliations:** † Department of Chemistry, University of Pavia, Via Taramelli 16, Pavia 27100, Italy; ‡ Department of Chemistry, Stephenson Institute for Renewable Energy, University of Liverpool, Liverpool L69 3ZD, U.K.; § National Reference Centre for Electrochemical Energy Storage (GISEL)—INSTM, Via G. Giusti 9, Firenze 50121, Italy

**Keywords:** nanoconfinement, lithium-ion batteries, metal−organic frameworks, dendrites, quasi-solid electrolytes

## Abstract

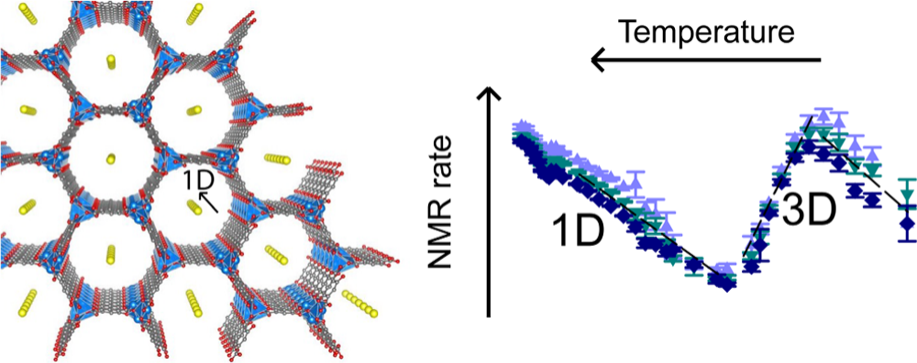

Metal–organic frameworks (MOFs) are excellent platforms to design hybrid electrolytes for Li batteries with liquid-like transport and stability against lithium dendrites. We report on Li^+^ dynamics in quasi-solid electrolytes consisting in Mg-MOF-74 soaked with LiClO_4_–propylene carbonate (PC) and LiClO_4_–ethylene carbonate (EC)/dimethyl carbonate (DMC) solutions by combining studies of ion conductivity, nuclear magnetic resonance (NMR) characterization, and spin relaxometry. We investigate nanoconfinement of liquid inside MOFs to characterize the adsorption/solvation mechanism at the basis of Li^+^ migration in these materials. NMR supports that the liquid is nanoconfined in framework micropores, strongly interacting with their walls and that the nature of the solvent affects Li^+^ migration in MOFs. Contrary to the “free’’ liquid electrolytes, faster ion dynamics and higher Li^+^ mobility take place in LiClO_4_–PC electrolytes when nanoconfined in MOFs demonstrating superionic conductor behavior (conductivity σ_rt_ > 0.1 mS cm^–1^, transport number *t*_Li^+^_ > 0.7). Such properties, including a more stable Li electrodeposition, make MOF-hybrid electrolytes promising for high-power and safer lithium-ion batteries.

## Introduction

Metal–organic frameworks (MOFs) are hybrid porous solids, obtained by reticular synthesis, through which inorganic metal ions or clusters are coordinated by organic linkers to form a multidimensional framework.^1^ Due to the high flexibility in terms of chemical nature, size, and geometry of the components, more than 90,000 MOF structures are available in the Cambridge Structural Database (CSD)^2^ as of March 2021.^3^ The organic unit typically possesses a ditopic or polytopic functionality (often carboxylate) which is anchored to metal ions or clusters to yield a (often) robust crystalline MOF structure with a porosity degree typically higher than 50%. By tuning the metal ions and linkers, ultrahigh porosity may be achieved with pore diameters in the micro–meso domain, namely, lower than 10 nm. This results in surface area values larger than 7000 m^2^ g^–1^ with a theoretical upper limit of 14,600 m^2^ g^–1^,^4^ exceeding those typically observed in other class of porous materials such as zeolites and porous carbons.^5^ In addition, MOF thermal and chemical stabilities allow postsynthesis functionalization, which enable their use in a wide range of applications, from gas storage, separation, and adsorption to catalysis and ion and/or electron conduction.^1^

Recently, MOFs emerged as a promising category of functional materials for electrochemical energy-storage and conversion technologies, more specifically as electrolytes in the case of Li- and post-lithium-ion batteries.^6,7^ The reason of such interest lies in the fact that MOF-based ion conductors could potentially fit some constraints on the new-generation electrolyte design imposed by the recent development of Li-metal batteries, such as high conductivity, chemical and electrochemical stability against the electrodes, and higher safety.^8^ Two other significant properties that characterize the electrochemistry of these systems: (i) high Li^+^ transference numbers *t*^+^, whose values can easily exceed 0.6;^9,10^ (ii) capability to block the formation of lithium dendrites, constituting a mechanically rigid barrier by themselves which favors uniform Li electrodeposition.^8,11^

Remarkable ion conduction has been demonstrated in functionalized MOFs, both under liquid-confined and anhydrous conditions.^8^ Very recently, a novel Cu(II)-azolate MOF (MIT-20) with open tubular pores was developed as a Li superionic solid electrolyte for Li-ion batteries.^12^ It was shown that by adding a stoichiometric amount of LiCl or Li pseudo-halide salts, this MOF undergoes a reversible single crystal–single crystal transition between neutral and Cl/pseudo-halide-anionic phases.^12,13^ This allows impressively high loading of charge-balancing alkaline metal ions for MOFs, which move within the 1D pores, giving single-ion conductivities for Li^+^ and Na^+^ higher than 0.01 mS cm^–1^ at room temperature and activation energy barriers *E*_a_ comprised between 0.16 and 0.32 eV for Li^+^, depending on the anion.^12^ Similar results were found in the case of other honeycomb-like structured MOFs with 1D channels along the *c*-axis, such as the cucurbit[6]uril-based derivatives, which exhibit conductivity of about 0.1 mS cm^–1^ and high cationic transference number (*t*^+^ > 0.7) after incorporation of LiPF_6_ into the pores.^14,15^

The ionic conductivity in MOF-based systems may be further enhanced using the MOF as a microporous and nanostructured scaffold for the encapsulation of liquid electrolytes. The resulting conductor is a “quasi-solid” electrolyte, combining conductivities very close to those of the liquid phase with the robustness and the stability of a solid matrix. This hybrid system is possible due to the MOF open porosity and high absorption capability, which make the framework a reservoir for liquid electrolytes.^11^ In this case, the mechanism of Li^+^ transport is based on the synergy between three phenomena: (i) liquid nanoconfinement in the microporous structure, (ii) capillary effects which enable an easy retention of the liquid phase into the rigid matrix, and (iii) coordination of the Li^+^ counterion to the MOF metallic center, allowing Li ion to hop from one site to another.^8^

Other MOF-based quasi-solid electrolytes were recently proposed in the literature and their ionic properties were discussed in terms of MOF chemistry/microstructure and nature of the incorporated liquid electrolytes (carbonate-based or ionic liquids, ILs).^11,16−20^ Specifically, it was observed that the metal center, pore size, dimensionality, and tortuosity remarkably affect both transport numbers and conductivities. By properly modulating these parameters, a new family of superionic conductors may be designed, the best of which exhibit room-temperature conductivity higher than 1 mS cm^–1^,^9,10^ activation energy lower than 0.25 eV, and a significantly enhanced transport number, which in some cases, exceeds 0.6. These properties allow superior cycling performance and stability resulting in limited capacity fading (typically lower than 70%) after a high number of charge/discharge cycles.^9^

The Li^+^ migration in MOF-based systems containing nanoconfined liquids is also largely affected by the interplay between the confinement effect and the interaction between the liquid electrolyte and the pore walls, especially in the case of ILs. This confers to the liquid phase very distinct ionic and physicochemical properties in the restricted space compared to the bulky material.^9,10,21,22^

Correlating the influence of different MOF pore environments on the Li-ion binding in the framework and Li^+^ dynamics is an important aspect in improving the design of MOF-based electrolytes and understanding the nature of the host–guest interactions.^23,24^ In order to provide more insights on the ion migration mechanism in these systems, it is of interest to determine the relative distribution of Li ions in nanosized MOF pores (in-pore) and in microsized voids (ex-pore) among the particles of the frameworks; this can be achieved by tuning important parameters such as the cage dimension and the nature of the liquid electrolyte (e.g., viscosity, concentration, solvents, and consequently, salt dissociation). Nuclear magnetic resonance (NMR) is fast becoming a powerful tool to probe in- and ex-pore species^25^ by distinguishing the interactions occurring in the nanopores and within voids among the particles and has recently been used to trace in-pore ion transport in UiO-66(Zr).^21^ In particular, NMR spin–lattice (SLR) rate measurements enable the understanding of the pore-type–structure–ionic diffusivity relationships in MOFs^23,24^ and other porous materials.^26−28^

The present work aims at investigating the Li^+^ dynamics in Mg-MOF-74-based quasi-solid electrolytes by combining conductivity and transport number measurements with NMR spectroscopy. Specifically, the aim of the work is to probe the confinement environments of the conductive liquid guest inside Mg-MOF-74 so as to characterize the related adsorption and solvation mechanism which are the basis of the lithium-ion migration in this unique functional material.

## Results and Discussion

### Mg-MOF-74: Structure and Morphology

**Figure 1:**
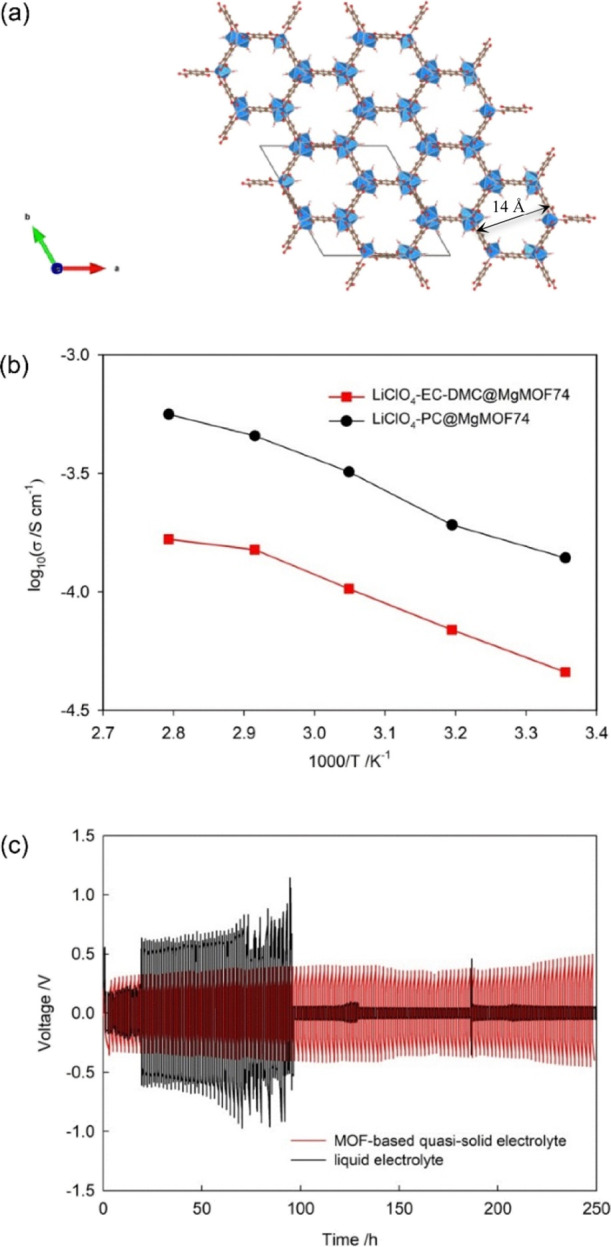
(a) Crystal structure of Mg-MOF-74, showing the hexagonal channels formed by Mg (blue) and the dhtp linker (carbons in gray, oxygens in red, and hydrogens omitted for clarity), running parallel to the crystallographic *c* axis. (b) Arrhenius plots of the ionic conductivity for the LiClO_4_-PC and LiClO_4_-EC-DMC@MgMOF74 quasi-solid electrolytes. (c) Li plating/stripping behavior of Li//Li symmetric cells with different electrolytes; black line: liquid LiClO_4_–PC 2.0M and red line: quasi-solid LiClO_4_-PC@MgMOF74.

Mg-MOF-74, also known as CPO-27-Mg, crystallizes in the trigonal space group *R*3̅ (n. 148).^29^ As shown in Figure 1a, its structure is characterized by channels running along the crystallographic *c* axis. In this structure, each metal in the cluster is octahedrally coordinated by five oxygen atoms belonging to the surrounding linkers, while the sixth position in each octahedron is occupied by a solvent molecule. Removal of the solvent molecules ensures the formation of unsaturated metal centers which provide charged binding sites that enhance the guest–host interaction for small molecules.^30^

Figure S1 shows the X-ray powder diffraction (XRPD) patterns of the as-prepared Mg-MOF-74 and the powder sample activated at a maximum temperature of 200 °C. The calculated XRPD pattern of Mg-MOF-74 is also reported for comparison.^31^ The agreement between the as-prepared and the simulated data indicates that the synthetic procedure adopted in this study is successful in crystallizing the desired material. Comparison between the XRPD pattern of the as-prepared and the activated sample supports the idea that the activation procedure under vacuum at a maximum temperature of 200 °C does not alter the long-range structural order in the sample. Small differences in the intensity ratio may be ascribed to different numbers of solvent molecules and absorbed water. Indeed, the simulated pattern of the material, once water is removed from the crystal structure, presents a different intensity ratio between relevant Bragg peaks, as shown in Figure S2a. Figure S2b shows a comparison between the XRPD patterns of the powder sample activated according to the two different procedures explored in this study. Both procedures allow us to maintain the long-range order of the structure, as indicated by the similarity between the XRPD patterns; however, inspection of the experimental results suggests that the activation procedure reported in the literature,^32^ requiring a higher temperature (*T*_max_ = 250 °C), produces a sample characterized by an XRPD pattern with slightly broader peaks, suggesting an increased amount of structural disorder. For this reason, the subsequent characterization was carried out on samples activated at a maximum temperature of 200 °C, which also required a shorter activation time.

As shown by BET measurements reported in Figure S3, Mg-MOF-74 exhibits a typical hierarchical microporosity with a pore size of about 2–4 nm (in good agreement with the X-ray studies), a surface area of 544 m^2^ g^–1^ and a volume of 0.30 cm^3^ g^–1^. The microstructure is fully retained after the properly controlled activation step, as confirmed by XRD analysis previously described. Figure S4 shows the scanning electron microscopy (SEM) images of the as-prepared Mg-MOF-74 sample at two different magnifications. The sample is constituted by well-distributed and homogeneous particles <1 μm; in some of them, the hexagonal shape is clearly visible.

### Mg-MOF-74–Based Quasi-Solid Electrolytes: Li-Ion Transport

The activated Mg-MOF-74 is an electric insulator. Very low conductivity values were determined by means of electrochemical impedance spectroscopy (EIS) (6.0 × 10^–11^ S cm^–1^), whose Nyquist plot is shown in Figure S5, reasonably due to the presence of impurities or a small amount of residual solvent.

The MOF was impregnated by two different liquid electrolytes, changing for concentration and solvents, namely, LiClO_4_ in propylene carbonate (PC) 2.0M and LiClO_4_ in ethylene carbonate (EC)/dimethyl carbonate (DMC) (1/1 v/v) 1.0M. The resulting hybrid electrolytes were selected among a number of compositions we investigated in screening steps by changing the solvent (PC and EC-DMC), the lithium salt (LiPF_6_, LiClO_4_, and LiTFSI), and solvent concentration (0.5, 1.0, and 2.0M for PC and 1.0M for EC-DMC). Table S1 compares the ionic conductivity at 25 °C of such systems, in which a maximum in conductivity is clearly observed for the two abovementioned MOF-based electrolytes. A direct comparison between MOF-LiClO_4_/PC 2.0M and MOF-LiClO_4_/EC-DMC 2.0M was not possible since the salt was found to be partially insoluble in EC-DMC for concentrations higher than 1.0M. The encapsulation step was carried out by mixing the activated MOF powder and proper amounts of liquid under slight heating condition to better disperse the liquid into the porous matrix.^16^ Similar uptakes were obtained for both the solutions that are very close to the MOF pore volume, suggesting a fully open porosity and the incorporation of LiClO_4_-based systems into the pores to give two quasi-solid electrolytes, labeled in the following as LiClO_4_-PC@MgMOF74 and LiClO_4_-EC-DMC@MgMOF74 (see Table 1).

**Table 1:** Volume Uptake of Both the Liquid Electrolytes *V*_l_ (cm^3^ g^–1^) Li-Ion Transport Number *t*^+^, Activation Energy for the Ionic Conduction *E*_a_ (eV), and Ionic Conductivity σ at 20 °C (S cm^–1^) of the LiClO_4_-PC and LiClO_4_-EC-DMC@MgMOF74 Quasi-Solid Electrolytes

Sample	*V*_l_ (cm^3^ g^–1^)	*t*^+^	*E*_a_ (eV)	σ_20°C_ (S cm^–1^)
LiClO_4_-PC@MgMOF74	0.33	0.71	0.22(1)	1.4 × 10^–4^
LiClO_4_-EC-DMC@MgMOF74	0.37	0.52	0.23(3)	4.6 × 10^–5^

**Figure 2:**
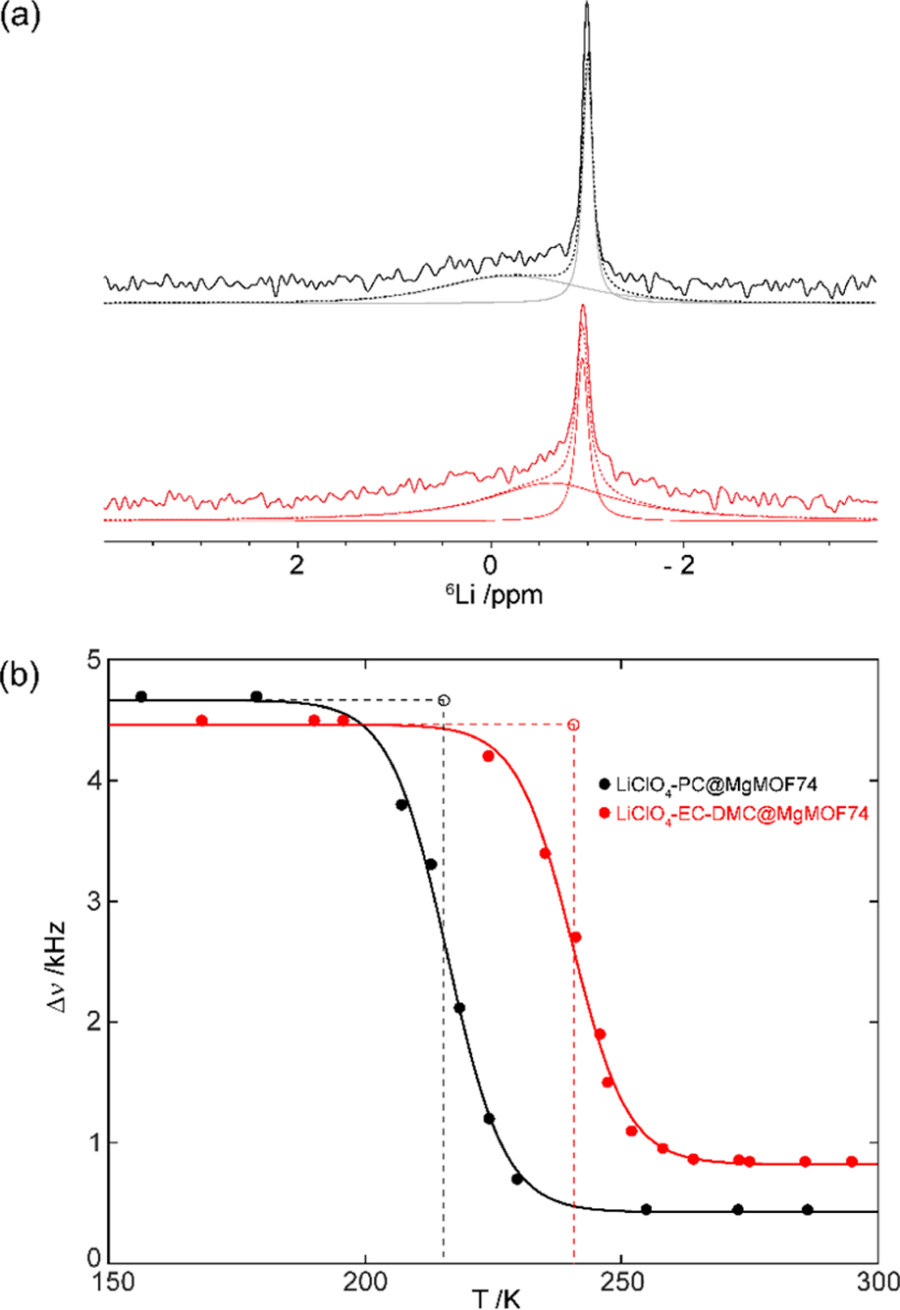
(a) ^6^Li MAS NMR spectra for LiClO_4_-PC@MgMOF74 (black) and LiClO_4_-EC-DMC@MgMOF74 (red). Experimental spectra, spectral deconvolution, and simulated spectra are shown with full, dashed, and dotted lines, respectively. (b) Temperature dependence of ^7^Li static NMR line width for LiClO_4_-PC@MgMOF74 (black) and LiClO_4_-EC-DMC@MgMOF74 (red). The Li^+^ jump rates τ^–1^ were determined by the dashed lines, which identify the temperature of the inflection point of the sigmoidal regression curve and NMR line width.^39^ A summary of the output of these fits is shown in Table S2.

In order to determine the ionic conductivity, the impregnated powder was pressed at 1 ton cm^–2^. The pellets were sandwiched between two stainless-steel plates and assembled in two-electrode cells for the EIS measurements. Selected examples of Nyquist plots are reported in Figure S6 with Figure 1b showing the Arrhenius plots for both the quasi-solid electrolytes in the temperature range between 20 and 70 °C. Despite LiClO_4_-EC/DMC 1.0M being a faster conductor (σ_rt_ = 8.5 mS cm^–1^) than the LiClO_4_-PC 2.0M one (5.5 mS cm^–1^),^33,34^ LiClO_4_-PC@MgMOF_74_ exhibits a higher room temperature conductivity than LiClO4-EC/DMC@MgMOF_74_ (0.14 mS cm^–1^ vs 0.046 mS cm^–1^, Table 1). This is only approximately 1 order of magnitude lower than the liquid PC-based solution (2.0M). The Li^+^ conductivities are thermally activated and hence expected to follow the Arrhenius behavior according to the well-known equation σ = σ_0_ exp(−*E*_a_/*k*_b_*T*) (Figure 2b) where σ_0_ is the pre-exponential factor and *k*_b_ is the Boltzmann’s constant. Of particular significance is the low activation energy obtained (*E*_a_ = 0.22 eV) for LiClO_4_-PC@MgMOF74 which is closer to the ones typically obtained for liquid rather than solid behaviors (typically 0.3–0.6 eV, sometimes even exceeding 1.2 eV).^35^ Considering the room-temperature conductivity in the order of 10^–4^ S cm^–1^ and the low *E*_a_ not higher than 0.25 eV, the system LiClO_4_-PC@MgMOF74 may be considered as a fast ion conductor.^36^ This behavior is in agreement with what recently observed in the case of several types of MOFs impregnated by different liquid electrolytes based on carbonates or ionic liquids as solvents.^9−11^ In particular, a lower conductivity measured for the liquid electrolyte entrapped in the MOF pores compared to the same liquid electrolyte solution is in line with previous results on liquid electrolytes encapsulated in nanoporous ceramic membranes^37^ and in MOF structures such as HKUST-1.^9^

In contrast, LiClO_4_-EC-DMC@MgMOF74 exhibits worse transport properties. Despite presenting a reasonably low activation energy of 0.24 eV, the room-temperature conductivity is only about 0.05 mS cm^–1^, more than 2 orders of magnitude lower than the corresponding solution. A possible reason for such a different behavior could lie in the different size of the solvents used. In the case of LiClO_4_-EC-DMC@MgMOF74, for instance, the solution is composed by a mixture of carbonates, one cyclic and one linear, that is overall more bulky than the single PC, which can affect, to some extent, the nanoconfinement of the liquid and modulate physical chemical properties of the solution, such as viscosity. As it is well-known, the viscosity of the solvent influences the conductivity of the liquid electrolyte solution, with higher conductivities usually associated to lower solution viscosities. This allows faster transport of ions in solution due to the inverse relation between ion mobility and viscosity represented according to the Stokes–Einstein equation.^33^ In addition, it has to be considered that the measured total conductivity is the result of the overall migration of both anions and cations, while for lithium batteries, only the portion of the current that is carried by the lithium cation contributes to effective battery cycling. This implies that rendering the anions immobile might correspond to an overall reduction in the total conductivity but not necessarily a reduction in the conductivity due to lithium. This is likely to have to be taken into account when comparing conductivity values of liquid electrolytes with their MOF-encapsulated counterparts.

We have also determined the cationic transport number, *t*_Li^+^_, of the two quasi-solid electrolytes using the Bruce–Evans methods, as described in the experimental section (see Figure S7 in Materials and Methods). Significantly high values, exceeding 0.5, were obtained for both matrices (Table 1), considerably higher than values reported for conventional carbonates systems. For comparison, *t*_Li^+^_ of 0.28 was reported for a liquid electrolyte composed of LiClO_4_ in PC^38^ and, in general, Li-ion transference numbers between 0.2 and 0.4 are reported for diluted nonaqueous Li solutions.^33^

A *t*_Li^+^_ value of 0.71 was in particular determined for the MOF encapsulating the LiClO_4_-PC based solution, even higher than the one recently obtained in the case of the HKUST framework imbibed by the similar LiClO_4_-PC 1.0M.^9^ The salt anion immobilization effect favoring the predominant Li ion transport is somewhat expected. A similar enhancement with respect to the liquid electrolyte was recently observed for several quasi-solid systems such as Al_2_O_3_ and ZrO_2_ and mostly MOFs.^8,37^ The ion-diffusion kinetics of MOF-modified systems incorporating liquid electrolytes was investigated by both spectroscopic and molecular dynamics simulations, suggesting that the anion migration is affected by two possible effects: (i) the coordination interactions with the MOF metallic center, significantly favored in the case of anions as ClO_4_^–^, Cl^–^ and TFSI^–^, becoming unsaturated during the activation step; (ii) the directional preference imposed by the MOF skeleton and the spatial restriction effects imposed by the well-ordered angstrom-scale pores on the anion transmission.^11^

The ClO_4_^–^ anion blocking within the MOF cages also has a stabilizing effect on the Li electrodeposition and, potentially, on the inhibition of the Li dendrite growth and proliferation. Figure 1c compares the Li plating/stripping behavior between the pristine electrolyte LiClO_4_-PC 2.0M and the corresponding MOF-based system impregnated by the same solution over more than 10 days. The experiments were performed in symmetric Li/electrolyte/Li cells that were cycled at a current density of 10 μA cm^–2^ with the corresponding areal capacity of 2.5 mA h cm^–2^. The voltage profile over prolonged cycling shows that the cell prepared with the quasi-solid electrolyte delivers a quite stable voltage plateau around 300 mV up to 250 operation hours with minimal overpotential. In contrast, the cell including the pristine liquid electrolyte exhibits a rapid increase in the overpotential after the 20 cycles, up to 500–600 mV, and drastic voltage fluctuations, due to the reaction between dendrites and the electrolyte itself, leading to cell abrupt short circuits. This result confirms the stabilizing role of the MOF-based system in the Li electrodeposition and its beneficial effect on the electrochemical performances of the Li metal cells, as recently reported in the literature for other MOFs with nanoconfined liquid electrolytes.^9−11,15^ Shen et al., for instance, showed a voltage profile of prolonged cycling in Li/MOF/Li symmetrical cells, including Zr-based UiO MOF or Cu-based HKUST systems imbibed with LiClO_4_-based electrolytes, delivering a stable voltage plateau (about 20 mV) upon long operation.^9^ Homogeneous electrodeposition was also observed in the case of NH_2_-MIL-125 (Ti) filled with 1.0M LiTFSI in 1,3-dioxolane/1,2-dimethoxyethane with 2% LiNO_3_, showing high Coulombic efficiency (99%) and good cycling stability in Li/Cu cells, due to positive effects of the amine groups in boosting the anode performance through their interactions with the ions in the electrolyte.^11^ The MOF capability to protect the Li metal from dendrite proliferation was also established by constructing the asymmetric solid polymer electrolyte modified by a robust layer of ZIF8. In this case, low and stable hysteresis over 800 cycles was maintained with high capacity retention after 100 cycles.

### Mg-MOF-74–Based Quasi-Solid Electrolytes: Li-Ion Dynamics

#### ^6^Li NMR Structural Characterization

^6^Li magic angle spinning (MAS) NMR experiments were performed at room temperature for LiClO_4_-PC@MgMOF74 and LiClO_4_-EC-DMC@MgMOF74 (Figure 2a) to probe the liquid nanoconfinement and, more specifically, the distribution of the adsorbed Li^+^ cations in the LiClO_4_ electrolyte solution into the ionic channels of Mg-MOF-74 (in-pore adsorbate) and/or nonadsorbed cations in the solution within the interparticle voids (ex-pore adsorbate). This approach has been previously used for the characterization of different MOF systems [e.g., UiO-66(Zr)], encapsulating small molecules such as acetone, methanol, or cyclohexane inside the framework pores, by obtaining possible perturbation of the chemical shifts occurring as a consequence of changes in self-solvation upon adsorption.^21^Figure 2a shows a sharp main peak at ∼−1.0 ppm in both NMR spectra as well as broad and poorly intense signals at ∼−0.6 and −0.2 ppm for LiClO_4_-EC-DMC@MgMOF74 and LiClO_4_-PC@MgMOF74, respectively. Based on the observed line widths, the broad ^6^Li signals were tentatively assigned to adsorbed Li^+^ cations in LiClO_4_ (in-pore adsorbate), while the sharp ^6^Li peaks were attributed to highly mobile and nonadsorbed Li^+^ cations (ex-pore adsorbate), indicating saturation of the MOF pores and filling the void space between crystallites.

#### ^7^Li NMR Line Narrowing

^7^Li static NMR spectra of LiClO_4_-EC-DMC@MgMOF74 and LiClO_4_-PC@MgMOF74 (Figure S8) were recorded as a function of temperature in order to access the mobility of the Li^+^ cations. At low temperatures, the ^7^Li NMR spectra are dominated by the strong homonuclear ^7^Li–^7^Li dipolar coupling interactions that broaden the 1/2 –1/2 central transition in the absence of lithium ionic mobility. In this rigid lattice regime, the corresponding ^7^Li NMR spectra display full width at half-maximum (fwhm) of ∼4.5 ± 0.2 kHz at 145 K for LiClO_4_-EC-DMC@MgMOF74 and of ∼4.8 ± 0.2 kHz at 134 K for LiClO_4_-PC@MgMOF74 (Figure S8). Upon heating, ^7^Li motional line narrowing is observed and largely results in the averaging of the ^7^Li dipolar coupling interaction due to thermally activated Li^+^ ion mobility (Figure 2b). The onset temperature for line narrowing, *T*_onset_, occurs at a lower temperature for LiClO_4_-PC@MgMOF74 (∼200 K) than in LiClO_4_-EC-DMC@MgMOF74 (∼225 K) and demonstrates faster Li^+^ dynamics in the former material containing PC than in the EC/DMC carbonate mixture. Using the Waugh–Fedin expression,^40^*E*_a_ = 1.67 × 10^–4^*T*_onset_, it is possible to extract an approximate of the activation energy by relating the onset temperature of motional narrowing with the activation energy of the diffusion process. Using this approach, activation energies of ∼0.3 and 0.4 eV can be estimated for LiClO_4_-PC@MgMOF74 and LiClO_4_-EC-DMC@MgMOF74, respectively. In the fast-motional regime, the ^7^Li central transition of the static NMR spectrum of LiClO_4_-EC-DMC@MgMOF74 exhibits a broader line (fwhm ∼ 0.85 kHz) than in LiClO_4_-PC@MgMOF74 (fwhm ∼ 0.50 kHz), also likely indicating higher Li^+^-ion mobility occurring in the latter electrolyte. Furthermore, comparison of the Li^+^ jump rate, τ^–1^ (which is in the order of the ^7^Li central transition NMR line widths in the rigid lattice regime and is quantified at the temperature of the inflection point, dashed lines in Figure 2b) for both electrolytes yields values of approximately ∼3 × 10^4^ s^–1^ at ∼240 and 216 K for LiClO_4_-EC-DMC@MgMOF74 and LiClO_4_-PC@MgMOF74, respectively. All these data indicate faster Li^+^ dynamics in LiClO_4_-PC@MgMOF74 than in LiClO_4_-EC-DMC@MgMOF74 and support the conductivity data (Table 1) discussed above.

#### Variable-Temperature ^7^Li NMR Spin Relaxometry

**Figure 3:**
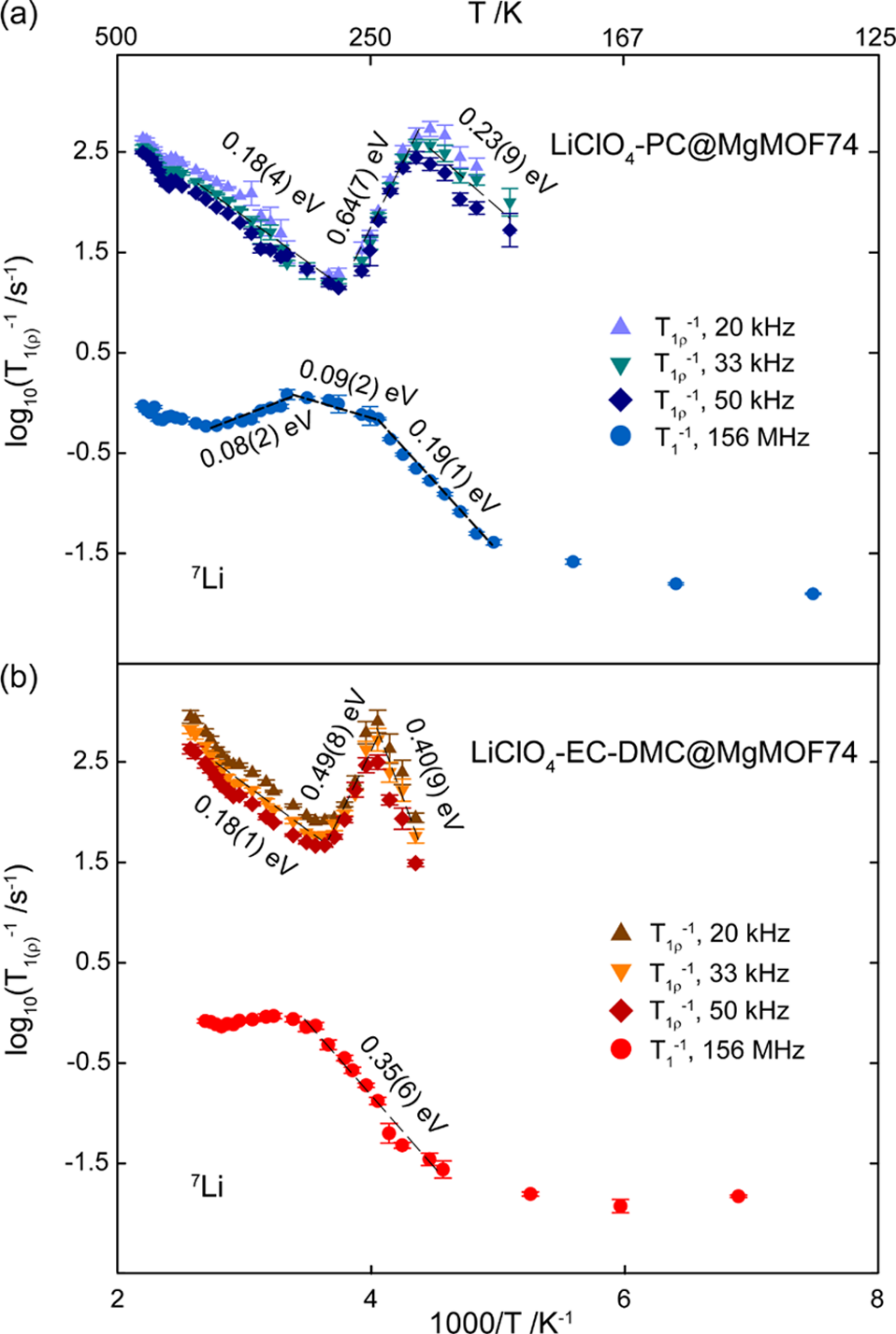
Arrhenius plots of the ^7^Li NMR SLR in the laboratory frame (T_1_^–1^) at ω_0_/2π = 156 MHz and in the rotating frame (*T*_1ρ_^–1^) at ω_1_/2π = 20, 33, and 50 kHz for (a) LiClO_4_-PC@MgMOF74 (blue) and (b) LiClO_4_-EC-DMC@MgMOF74 (red). The temperature range used to determine activation barriers *E*_a_ is represented by black dotted lines.

Temperature dependence of ^7^Li NMR SLR rate measurements in the laboratory frame (T_1_^–1^) and rotating frame (*T*_1ρ_^–1^) under static conditions provides useful information on the Li^+^ dynamics in the scale of the Larmor (ω_0_/2π = 156 MHz, see Materials and Methods) and spin-lock (ω_1_/2π = 20, 33 and 50 kHz) frequencies, respectively. Arrhenius behavior of the correlation times τ is observed and the slopes of the high- and low-temperature regions of the SLR rates are used to determine the activation energies for the Li-ion diffusional process, where the low- and high-temperature slopes correspond to short- and long-range motional processes, respectively. In LiClO_4_-PC@MgMOF74 (Figure 3), SLR T_1_^–1^ values increase upon heating from 134 K and pass through a maximum at 286 K before decreasing, yielding an activation barrier of 0.19(1) eV in the low-*T* limit (ω_0_τ_c_ ≫ 1, where τ_*c*_ is the correlation time of Li^+^ motion) and 0.09(2) eV between 247 K and 286 K. Similarly, in the high-*T* limit (ω_0_τ_*c*_ ≪ 1), the data retain an activation energy of 0.08(2) eV. In LiClO_4_-EC-DMC@MgMOF74 (Figure 3b), the data yield an activation energy of 0.35(6) eV up to 309 K, in which a maximum of SLR T_1_^–1^ values was observed.

Similarly, plots of the SLR T_1ρ_^–1^ values as a function of temperature show an increase in *T*_1ρ_^–1^ values as temperature increases in the slow motional regime (ω_1_τ_c_ ≫ 1) with activation barriers of 0.23(9) eV between 196 K and 224 K for LiClO_4_-PC@MgMOF74 (Figure 3a) and 0.40(9) eV for LiClO_4_-EC-DMC@MgMOF74 (Figure 3b). The plots of SLR *T*_1ρ_^–1^ values as a function of temperature show the presence of maxima at 224 K for ω_1_/2π = 20 and 33 kHz and at 230 K for ω_1_/2π = 50 kHz for LiClO_4_-PC@MgMOF74 and a single *T*_1ρ_^–1^ maximum at 247 K for all spin-lock frequencies in LiClO_4_-EC-DMC@MgMOF74. Accessing these *T*_1ρ_^–1^ maxima enables NMR-derived correlation rates, τ_c_^–1^, to be obtained, as these maxima occur at temperatures that are characteristic of the Li correlation rates τ_c_^–1^ being proportional to the spin-lock frequencies, that is, ω_1_τ_*c*_ ≈ 0.5 (Figure 3). The lower temperature of the *T*_1ρ_^–1^ maximum for LiClO_4_-PC@MgMOF74 versus LiClO_4_-EC-DMC@MgMOF74 further confirms faster Li^+^-ion mobility in the former material in line with the ^7^Li static NMR lineshape above and conductivity data. At temperatures above the *T*_1ρ_^–1^ maxima, in the fast motional regime (ω_1_τ_c_≪1), the activation energies provided by the high-T flank and dominated by diffusion processes are significantly larger than the values extracted by the Arrhenius plot of the impedance data (0.22 eV for LiClO_4_-PC@MgMOF74 and 0.21 eV for LiClO_4_-EC-DMC@MgMOF74).

We note that the lack of resolution of the ^7^Li static NMR spectra does not allow for the separation of the ^7^Li signals arising from the immobile adsorbed (in-pore adsorbate) and mobile nonadsorbed (ex-pore adsorbate) Li^+^ cations in LiClO_4_ detected in the ^6^Li NMR data. It is therefore postulated that the NMR data capture the motion of both Li^+^ cations and that the activation energies obtained are a weighted-average of the mobile and immobile Li^+^ that would have different activation energies from the ones obtained from the ionic conductivity data that only measures the fast-moving low activation energy Li^+^ ions. As the temperature is increased even further (>260 K), both materials show increasing values of *T*_1ρ_^–1^ once more and with equal activation barriers of 0.18 eV for LiClO_4_-EC-DMC@MgMOF74) and LiClO_4_-PC@MgMOF74, respectively. This is a clear indication of the presence of more than one diffusion process; however, it was not possible to fully probe this second diffusion process as increasing the temperature any further would have led to sample degradation. Due to the higher temperature at which these *T*_1ρ_^–1^ maxima occur, it is highly likely that this diffusion process is due to the slower 1D diffusion inside the Mg-MOF-74 channels, while the initial *T*_1ρ_^–1^ maxima observed at 224 and 230 K for LiClO_4_-PC@MgMOF74 and 247 K for LiClO_4_-EC-DMC@MgMOF74 can be attributed to the highly mobile Li diffusion between crystallites.

The SLR rate data in Figure 3 show an asymmetric behavior and therefore indicate deviation from the quadratic dependency of the rates with respect to reciprocal temperature predicted by Bloembergen-Purcell-Pound (BPP) theory of relaxation (i.e., *T*_1ρ_^–1^ ∝ ω^–β^ where β = 2) as this is often the case in fast Li-ion conductors. This is likely due to correlation effects (e.g., Coulombic interactions) often found in complex and/or disordered fast ion conductors that yield a range of short- and long-range motional processes. This behavior can be quantified from the spectral density function that yields the following expression *E*_a,low_ = *E*_a,high_(β −1), where β, *E*_a,low_, and *E*_a,high_ are the correlation factor and the activation barrier in the slow and in fast motional regimes, respectively. If β < 2, asymmetric BPP curves are observed indicating the presence of correlation effects. In this work, β values of ≈1.4 and ≈1.8 for LiClO_4_-PC@MgMOF74 and LiClO_4_-EC-DMC@MgMOF74 were calculated, respectively. This result is expected due to the increased concentration of LiClO_4_ in LiClO_4_-PC@MgMOF74 (2M) compared to LiClO_4_-EC-DMC@MgMOF74 (1M), leading to an increased degree of Coulombic interactions.

**Figure 4:**
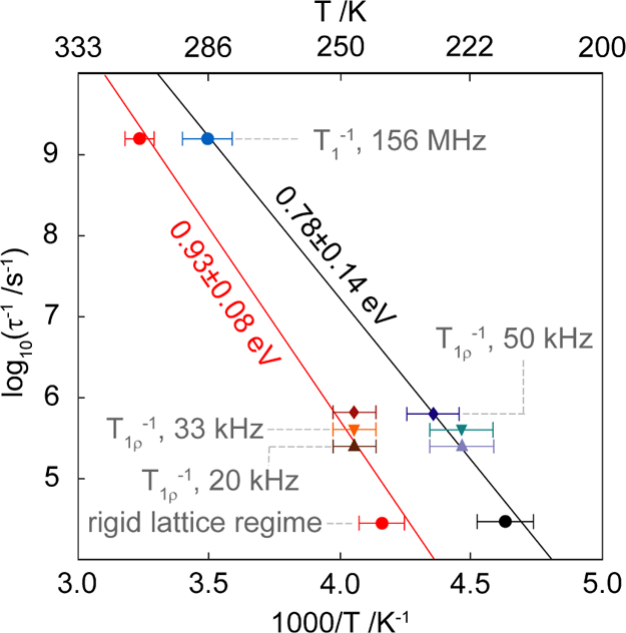
Arrhenius plot of Li^+^ jump rates τ ^–1^ obtained from ^7^Li NMR line width plot and NMR SLR measurements for LiClO_4_-PC@MgMOF74 (black) and LiClO_4_-EC-DMC@MgMOF74 (red). The jump rates were extracted from the line narrowing experiments in Figure 2b and the position of the maxima in the temperature dependency of the SLR rates *T*_1_^–1^ and *T*_1ρ_^–1^ data in Figure 3.

As mentioned previously, temperature-dependent Li^+^ jump rates τ^–1^ can be obtained from the inflection point of the ^7^Li NMR line width in the static regime (Figure 2b) and the position of the SLR *T*_1ρ_^–1^ and *T*_1ρ_^–1^ rate maxima (Figure 3). These Li^+^ jump rates τ^–1^ are thermally activated and hence expected to follow the Arrhenius behavior which fits reveal *E*_a_s of 0.79 ± 0.14 and 0.93 ± 0.08 eV for LiClO_4_-PC@MgMOF74 and LiClO_4_-EC-DMC@MgMOF74, respectively (Figure 4).

The dimensionality of Li^+^-ion mobility can be accessed from the frequency dependence of the high-temperature flanks of the SLR *T*_1ρ_^–1^ values and has been shown to follow characteristic relationships with one-, two-, and three-dimensional mobility in solids being proportional to (τ/ω_1_)^0.5^, τln (1/ω_1_τ), and τ, respectively,^41,42^ for which the corresponding fits are shown at various temperatures in the fast motional regime (Figures S9, S10). Quicker correlation times τ_c_ (≈10^–8^ s) than the kHz time scale indicate three-dimensional lithium diffusion.^40,41^ Therefore, the initial *T*_1ρ_^–1^ maxima observed at 224 and 230 K for LiClO_4_-PC@MgMOF74 and 247 K for LiClO_4_-EC-DMC@MgMOF74 can be attributed to the highly mobile 3D Li-ion diffusion outside the pores, as evidenced from *T*_1ρ_^–1^ values being independent of ω_1_, whereas the increase in *T*_1ρ_^–1^ (≫260 K) may be assigned to the low-*T* limit for the slower 1D diffusion inside the Mg-MOF-74 channels. The activation energies extracted through the various spectroscopic methods applied here are summarized in Table S3. Activation energies of 0.22(1) and 0.23(3) eV are determined through impedance spectroscopy for LiClO_4_-PC@MgMOF74 and LiClO_4_-EC-DMC@MgMOF74, respectively. Values of 0.19(1) and 0.35(6) eV are observed for the low-temperature flanks of the SLR *T*_1_^–1^ rates for LiClO_4_-PC@MgMOF74 and LiClO_4_-EC-DMC@MgMOF74, respectively. These values are indicative of the slow motion regime (where ω_0_τ_c_ ≫ 1), characterizing local hopping processes between local energy minima and unsuccessful jumps to the neighboring sites. Figure 3 displays two different Li^+^ diffusion processes, corresponding to 1D translational Li^+^ diffusion along the *c*-axis within the MOF pores and 3D Li^+^ diffusion between the crystallites. For the 3D diffusion, the low-temperature flank of the BPP curves yields values of 0.23(9) and 0.40(9) eV for LiClO_4_-PC@MgMOF74 and LiClO_4_-EC-DMC@MgMOF74, indicating a more favorable local hopping process found in the PC phase. The high-temperature flanks of the BPP curves, where ω_1_τ_c_ ≪ 1 corresponds to translational diffusion of Li^+^ ions, display activation barriers of 0.64(7) and 0.49(8) eV for LiClO_4_-PC@MgMOF74 and LiClO_4_-EC-DMC@MgMOF74, respectively, suggesting that the long-range 3D ion diffusion is largely similar in both phases. For the 1D diffusion within the MOF pores, only the low-temperature flank can be obtained in the available temperature range and corresponds to local Li^+^ hops within the MOF channels. The same values of 0.18(4) and 0.18(1) eV are derived for LiClO_4_-PC@MgMOF74 and LiClO_4_-EC-DMC@MgMOF74, respectively, indicating that the local hopping process in the pores of both materials is similar. Overall activation energies for both phases obtained from combining jump rates τ^–1^ extracted from SLR and motional narrowing NMR experiments are displayed in Figure 4. Values of 0.78(14) and 0.93(0.08) eV are obtained for LiClO_4_-PC@MgMOF74 and LiClO_4_-EC-DMC@MgMOF74, respectively, indicating a lower activation barrier for diffusion in the former phase. While there are discrepancies between the activation energies extracted from NMR compared with ACIS, this observation is likely due to the different time scales of the methods used, as NMR spectroscopy determines the barrier for diffusion of Li to neighboring sites, whereas impedance measurements probe longer-range translational Li^+^ diffusion.^23,43,44^

### ^13^C NMR Structural Characterization

**Figure 5:**
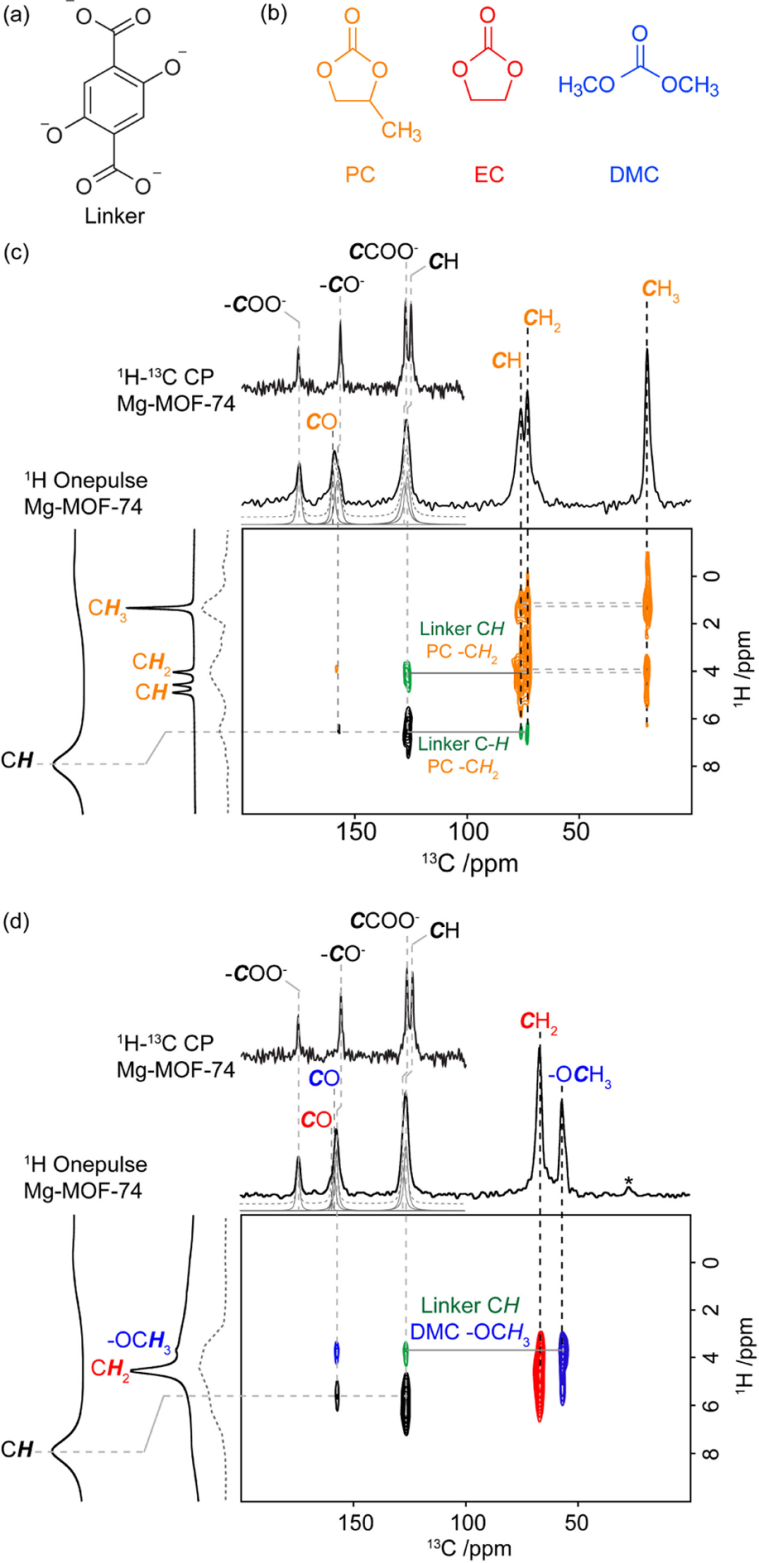
Chemical structures of (a) linker and (b) PC, EC, and DMC electrolytes. 2D ^1^H–^13^C HETCOR NMR spectra for (c) LiClO_4_-PC@MgMOF74 and (d) LiClO_4_-EC-DMC@MgMOF74 using a long CP contact time of 1 ms. Signals for the linker, PC, EC, and DMC are labeled in black, orange, red, and blue, respectively. The spectra for LiClO_4_-EC-DMC@MgMOF74 and LiClO_4_-PC@MgMOF74 show typical correlations for EC (in red), DMC (in blue), PC (in orange), and the MOF organic linker (in black). The extra correlations observed in these spectra versus the one obtained with a shorter CP contact time of 50 μs (Figure S12) are given in green to emphasize close proximity between the solvent and MOF linker (Figure S13), suggesting absorption into the pores. In the ^13^C direct dimension of the HETCOR, ^13^C CP MAS spectra of Mg-MOF-74, LiClO4-PC@MgMOF74 [(in panel (c)], and LiClO4-EC-DMC@MgMOF74 [in panel (d)], simulated (dashed gray lines) and deconvoluted spectra (gray lines) are given and show the shifts in the NMR signals of most carbons in the organic linker versus pure, thermally activated Mg-MOF-74. In the ^1^H indirect dimension, a comparison of the ^1^H one pulse spectra of Mg-MOF-74, the quasi-solid electrolytes (full lines), and the internal projections of the HETCOR (dashed gray lines) is provided from left to right. The asterisk (*) denotes the spinning sideband.

In order to further understand possible carbonates/MOF interactions, ^13^C MAS NMR spectra under a number of conditions (directly excited ^13^C one pulse spectra with and without ^1^H decoupling and with ^1^H–^13^C CP spectra at various contact times) were recorded (Figures 5, S11–S13). The ^13^C MAS NMR spectrum of the Mg-MOF-74 (on top of each panel in Figure S11, linker signal labels are given in black) exhibits four resonances at 174.6, 155.5 126.3, and 123.8 ppm corresponding to the −**C**OO^–^, −**C**O^–^, −**C**–COO^–^, and −**C**H linker signals, respectively supported by the previous literature^45^ and by the ^1^H–^13^C CP spectra carried out at different CP contact times in order to highlight directly protonated and quaternary carbons (Figure S12). The proposed ^13^C assignments for the LiClO_4_-PC@MgMOF74 and LiClO_4_-EC-DMC@MgMOF74 (Figure S11) are based on the Mg-MOF-74 ^13^C assignment, while the attribution of the peaks corresponding to liquid electrolytes (PC, EC, and DMC) is based on well-established values for ^13^C chemical shifts.^46^ Signal labels for PC, EC, and DMC are given in orange, red, and blue, respectively. Table S4 summarizes the ^13^C NMR spectral assignments of chemical shifts for all the samples studied in this work. ^1^H spectra for Mg-MOF-74 and for both the quasi-solid systems are given in Figure 5 on the left side of the respective HETCORs. The ^1^H Mg-MOF-74 spectrum exhibits a single broad peak corresponding to the aromatic MOF linker C–H groups (∼8 ppm). The ^1^H spectra of the quasi-solid systems are dominated by sharp peaks that can be attributed to the respective ^1^H electrolyte signals (PC for LiClO_4_-PC@MgMOF74 and EC-DMC for LiClO_4_-EC-DMC@MgMOF74)^46^ which overlap the broad aromatic MOF linker C–H group signal (green spots in Figure 5). The very broad linker ^1^H signal in the LiClO_4_-EC-DMC@MgMOF74 ^1^H spectra indicate solid components present in the sample or strongly adsorbed species into the pore in contrast to the narrow ^1^H resonances in the LiClO_4_-PC@MgMOF4. This suggests an increased quasi-solid behavior of the LiClO_4_-EC-DMC@MgMOF74 with respect the LiClO_4_-PC@MgMOF74 supporting the reduced Li conductivity found for the LiClO_4_-EC-DMC@MgMOF4.

^1^H–^13^C CP MAS NMR might provide excellent evidence of the presence of adsorbed molecules into the pores of the MOFs as recently illustrated on poly(ethylene oxide) (PEO) into UiO-66 with varying PEO content.^47^ A comparison of the ^13^C CP MAS NMR spectra of thermally activated Mg-MOF-74 with either LiClO_4_-PC@MgMOF74 or LiClO_4_-EC-DMC@MgMOF74 (Figure S11) shows shifts to lower frequencies in the ^13^C peak positions of most carbons in the quasi-solid system. In particular, shifts of about 2 ppm for the aromatic MOF linker C–H groups (from ∼126 ppm in Mg-MOF-74) for both LiClO_4_– PC@MgMOF74 and LiClO_4_-EC-DMC@MgMOF74 are observed suggesting close interactions with the nonaqueous solvent. For both samples, the intensity of the peaks at around 174 ppm largely increases when the CP contact time increases from 50 us to 5 ms (Figure S12), while the intensity for the peak at ∼130 ppm slightly increases when the CP contact time increases, supporting the ^13^C assignments that have been proposed (Table S4). The carbonyl resonances of the MOF linker at ∼174 ppm remain largely unchanged between Mg-MOF-74 and the quasi-solid systems presumably because the carboxylic group is more involved in bonding to the metal center rather than be oriented toward the ionic channels.

To further probe spatial proximity between solvent molecules and MOF, two-dimensional ^1^H–^13^C NMR HETCOR experiments were recorded with two different CP contact times on LiClO_4_-PC@MgMOF74 and LiClO_4_-EC-DMC@MgMOF74 (Figures 5 and S13). While spectra obtained with a short CP contact time of 50 μs only provide correlations in protonated carbons, additional correlations at a longer CP contact time (1 ms) indicate longer interactions (e.g., between the linker carbonyl signal at 126 ppm with the aromatics ^1^H at 6 ppm). In particular, correlations in green (Figure 5) between the ^13^C of the aromatic C–H (126 ppm) of the organic linker and the methylene ^1^H of PC (4 ppm), likewise proximity between the ^13^C of PC–CH_2_ (73 ppm) and the ^1^H of the aromatic C–H (6.6 ppm) in LiClO_4_-PC@MgMOF74 and between the aromatic carbon and the methoxy ^1^H of DMC (3.7 ppm) in LiClO_4_-EC-DMC@MgMOF74, are observed and confirm adsorbate-MOF close contact and the presence of in-pore liquid electrolytes.

## Conclusions

Quasi-solid electrolytes based on MOF-Mg-74 were prepared by soaking them in two different liquid electrolytes, namely, LiClO_4_ 2M in PC and LiClO_4_ 1.0M in EC-DMC. The effect of the solution nanoconfinement on the Li-ion dynamics is discussed for the first time in this kind of MOF. To this aim, electrochemical tests and a full solid-state NMR structural characterization were performed, which demonstrate that the chemical nature of the solvents strongly impact the ion mobility. Despite the lower conductivity of “free” LiClO_4_-PC than LiClO_4_-EC/DM pristine solutions, significantly better ionic transport properties are obtained when the PC-based electrolyte is confined in the MOF, showing higher conductivity at room temperature (>0.1 mS cm^–1^) and higher lithium transport number (*t*^+^ = 0.71).

^6^Li NMR spectra revealed that the liquid distribution through the MOF structure occurs by saturating the framework ionic channels (in-pore adsorbate) and the interparticle voids (ex-pore adsorbate), with further evidence of the presence of absorbed molecules in the MOF pores provided by ^13^C CP MAS NMR. The ^7^Li NMR relaxometry data suggest two possible Li^+^ diffusion processes, namely, the slower 1D diffusion inside the Mg-MOF-74 channels and the highly mobile Li diffusion between crystallites. The lower onset temperature for ^7^Li motional line narrowing detected and the sharper ^7^Li central transition line in LiClO_4_-PC@MgMOF74 than in LiClO_4_-EC-DMC@MgMOF74 demonstrate faster dynamics in the former material which is further strengthened by comparing the Li^+^ jump rate, τ^–1^. Such differences may be interpreted taking into account the presence of solid components or strongly adsorbed species into the pore, indicated by ^1^H spectra of LiClO_4_-EC/DMC@MgMOF74, in contrast to the narrow ^1^H resonances in the PC-based hybrid electrolyte. This suggests that the LiClO_4_-mixed carbonate solution behaves like a quasi-solid when it is nanoconfined in the porous MOF, contrary to the LiClO_4_-PC in liquid, supporting the decreased Li conductivity found for the LiClO_4_-EC-DMC@MgMOF4.

## Supporting Information Available

Materials and methods; additional XRPD patterns; isotherms; SEM images; Nyquist plots; chronoamperometry and EIS data; ^7^Li static NMR spectra; ^7^Li SLR Arrhenius plots; ^7^Li SLR *T*_1ρ_^–1^ frequency dependence plots; and additional ^13^C NMR spectra (PDF)
