# Supplementary material for: Li+ Dynamics of Liquid Electrolytes Nanoconfined in Metal–Organic Frameworks
Source: ACS Appl Mater Interfaces. 2021 Nov 9;13(45):53986–95. doi: 10.1021/acsami.1c16214 (PMC8603352; doi:10.1021/acsami.1c16214)
Supplement: Supplementary file 1 — am1c16214_si_001.pdf [file am1c16214_si_001.pdf]

Supplementary Materials for

Li^+ dynamics of liquid electrolytes nanoconfined in metal organic frameworks

Marco Farina[a#], Benjamin B. Duff[b#], Cristina Tealdi[a,c], Andrea Pugliese[b], Frédéric Blanc[b,*] and Eliana Quartarone[a,c*]

[a] *Department of Chemistry, University of Pavia, Via Taramelli 16 27100, Pavia, Italy*

[b] *Department of Chemistry, Stephenson Institute for Renewable Energy, University of Liverpool, Liverpool L69 3ZD, United Kingdom*

[c] *National Reference Centre for Electrochemical Energy Storage (GISEL) - INSTM, Via G. Giusti 9, 50121 Firenze, Italy*

**Corresponding author:* frederic.blanc@liverpool.ac.uk; eliana.quartarone@unipv.it

[#] *These authors contributed equally*

This PDF file includes:

Materials and Methods

Figs. SI1 to SI14

Table SI1 e SI2

References

Materials and Methods

General considerations

All the chemicals used in this work were purchased from Aldrich and used without further purification. All the operations regarding the preparation of the quasi-solid electrolytes, the cell assembly for the electrochemical tests, as well as the packing of the NMR samples in HRMAS inserts, were carried out in an Ar-filled glove box (MBraum, H_2O, O_2 < 1ppm).

Synthesis of Mg-MOF-74 and preparation of the Li@Mg-MOF-74 quasi-solid electrolyte

Mg-MOF-74 was prepared according to the green synthetic procedure previously described in the literature.[1] An aqueous solution of $Mg(NO_3)_2 \cdot 6H_2O$ (purity>99%) was rapidly added to an aqueous solution of dhtp (2,5-dihydroxyterephtalic acid) (purity>98%)and NaOH under stirring with a Mg/dhtp/NaOH molar ratio of 2:1:4 in a total volume of 10 mL. The reaction mixture was stirred for 4 hours at room temperature. A quick colour change from a transparent to yellow solution was clearly visible, indicating that the reaction is starting. The resulting yellow powders were collected by centrifugation at 6000 rpm for 20 minutes, washed three times with distilled water and methanol, and dried at 70°C overnight.

As-synthesised Mg-MOF-74 powders (200 mg) were activated by solvent exchange with methanol (10 mL) for 12 days (with daily methanol replacement) followed by a drying step in an alumina boat crucible under vacuum (10^{-5} bar) in a tubular furnace to yield a light-yellow powder. Using heating and cooling ramp rates of 5°C min^{-1}, the sample was either heated to 80°C, 100°C, 150°C, 200°C and held for 1 hour and then to 250 °C for 2 hours followed by cooling down to room temperature[1] or, in a second procedure tested in this work, heated to 200°C and held for 12 hours before cooling down.

The MOF-based quasi solid electrolytes were obtained by impregnation of Mg-MOF-74 with $LiClO_4$ in liquid electrolytes using either propylene (PC) 2.0M or ethylene:dimethyl carbonates (EC:DMC) (1:1 v:v) 1.0M through capillary action. In a typical procedure, activated Mg-MOF-74 (75 mg) was soaked in a mortar with 45 μL of either liquid electrolyte solution and thoroughly mixed with a pestle until a colour change was observed from light yellow to a darker yellow. In order to ensure a better dispersion of the liquid electrolyte into the pores, the Li@Mg-MOF-74 sample was then heated at 40°C for 1 hour. The amount of liquids confined in the system was calculated in terms of volume uptake and determined to saturate the pores. Taking into account the BET analysis, the Mg-MOF-74 pore volume, V_p, results to be 22.5 μL. The final loading amounts of the liquid electrolyte, V_l, were 24.6 μL in case of $LiClO_4$ 2.0M (d_{sol} = 1.42 g cm^{-3}) and 27.5 μL for $LiClO_4$-

EC/DEC 1.0M (d_{sol} = 1.01 g cm^{-3}). Since the final loading values are higher than pore volume for each sample, we assume all pores are saturated, while the excess is ex-pore.

Structural and morphological characterization

X-ray powder diffraction (XRPD) patterns of the as-prepared and activated Mg-MOF-74 samples were acquired at room temperature between 3° and 40° degrees inside an Ar-filled sample holder by using a Bruker diffractometer D8 Advance with a monochromatic Cu source (Kα1, λ = 1.5406 Å) in Debye-Scherrer geometry.

SEM analyses on MOF were performed using a Tescan Mira3XMU microscope operated at 20 kV and equipped with an EDAX EDS analysis system. The sample was coated with a carbon thin film using a Cressington 208 carbon coater.

N_2 adsorption-desorption analysis was performed with a Coulter SA 3100 instrument. The surface area was calculated by Brunauer Emmet Teller (BET) method. The pore size distribution was determined by Barrett-Joyner-Halenda (BJH) method.

Electrochemical measurements on Li@Mg-MOF-74

The ionic conductivity was measured between 20°C and 70°C by means of Electrochemical Impedance Spectroscopy (EIS), using a Frequency Response Analyzer (FRA) (Solartron 1255) connected to an Electrochemical Interface (Solartron 1287), by applying an AC voltage of 100 mV in the frequency range between 0.1 Hz-10^5 Hz. The experiments were carried out on 600 µm thick-pellets (diameter: 1 cm) obtained by pressing the impregnated MOF powders at 1 ton for 1 min. The sample was left equilibrating at each temperature for 1 h.

The Li transference number, t^+, was calculated by coupling EIS and chronoamperometry experiments on a Li/Li@MOF/Li symmetrical cell, as defined by the following equation:[2]

$$t^+ = \frac{I^{SS}(\Delta V - I^0 R^0)}{I^0(\Delta V - I^{SS} R^{SS})} \qquad \text{(S1)}$$

where ΔV is the applied voltage (15 mV), I^0 and I^{ss} are the current densities at the beginning of the polarization and at the steady state, respectively, and R^0 and R^{ss} are the interfacial resistances before and after polarization, respectively. High Li ion transference number, ideally close to 1, is a much desired property for electrolytes, as a low transference number implies large movement of the anions, which would cause concentration polarization during battery operation.

The Li electrodeposition was studied by means of stripping/plating experiments performed at room temperature on Li/Li@Mg-MOF-74/Li symmetric cells, assembled using a Swagelok and using an Arbin (model BT-2000) battery cycler. A small amount of PolyVinylidene Fluoride (PVDF) was added in the mortar during the preparation of the MOF-based quasi solid electrolyte to make the electrolyte pellet more processable for the subsequent characterization. The cell was cycled at a fixed current density of 0.01 mA cm^{-2} and plated/stripped for 1 hour during each cycle. For comparison, a symmetrical Li/$LiClO_4$-PC-2M/Li cell was also prepared using a Whatman separator and tested under the same experimental conditions.

Solid State NMR

^{6}Li magic angle spinning (MAS) NMR spectra were recorded at a Larmor frequency of $\omega_0/2\pi$ = 60 MHz using a 4 mm HXY MAS probe (in double resonance mode) on a 9.4 T Bruker DSX solid-state NMR spectrometer. All data acquisitions were quantitative using recycle delays longer than five-times the spin-lattice relaxation times T_1 (measured using a standard saturation recovery sequence). ^{6}Li NMR data were obtained with a pulse of length 3 µs at a radio frequency (rf) field amplitude of $\omega_1/2\pi$ = 83 kHz and at a MAS rate of $\omega_r/2\pi$ = 8 kHz. The ^{6}Li NMR spectra were referenced to 10 M LiCl in D_2O at 0 ppm.

^{7}Li static NMR spectra were recorded at $\omega_0/2\pi$ = 156 MHz using a 4 mm HXY MAS probe (in double resonance mode) below room temperature and a 4 mm HX High Temperature MAS probe above room temperature on a 9.4 T Bruker Avance III HD spectrometer. All ^{7}Li NMR spectra were obtained with a pulse length of 1.5 µs at a rf amplitude of $\omega_1/2\pi$ = 83 kHz. Spin-lattice relaxation rates in the laboratory frame (T_1^{-1}) were obtained using a saturation recovery pulse sequence and the data fitted to the stretch exponential function $1-\exp[-(\tau/T_1)^{\alpha}]$ where τ is the variable delay and α the stretch exponent factors (fitted α values between 0.6 and 1 were extracted). These were used in order to account for a distribution of correlation times as well as temperature gradients across the sample. Spin-lattice relaxation rates in the rotating frame ($T_{1\rho}^{-1}$) were obtained using a standard spin-lock pulse sequence at ^{7}Li frequencies $\omega_1/2\pi$ of 20, 33 and 50 kHz, fitting the data to a stretch exponential function of the form $\exp[-(\tau/T_{1\rho}^{-1})^{\beta}]$ (fitted β values ranging from 0.3 and 1 were obtained). The temperature calibration of the probes were performed using the ^{207}Pb NMR chemical shift thermometer of $Pb(NO_3)_2$.[3,4] The standard error associated with this method arises from temperature gradients across the sample which ranged from 2-7 K and were determined experimentally through the peak width of the static powder pattern of $Pb(NO_3)_2$. All ^{7}Li NMR spectra were referenced to 10 M LiCl in D_2O at 0 ppm. No ^{1}H decoupling was applied during any ^{6,7}Li NMR experiments.

All ^{1}H and ^{13}C MAS NMR spectra were recorded at Larmor frequencies of $\omega_0/2\pi$ = 400 and 100 MHz, respectively, using a 4 mm HXY MAS probe (in double resonance mode) on Bruker Avance III HD NMR spectrometer under MAS condition at a frequency $\omega_r/2\pi$ = 10 kHz at room temperature. All ^{1}H pulses were performed at a rf amplitude of $\omega_1/2\pi$ = 83 kHz. The ^{1}H-^{13}C cross polarization (CP) and heteronuclear correlation (HETCOR) spectra were acquired using ^{13}C rf field amplitude of $\omega_1/2\pi$ = 50 kHz matched to obtain maximal signal at a ^{1}H rf field of $\omega_1/2\pi$ = 60 kHz, with a SPINAL-64 heteronuclear decoupling[5] rf amplitude during ^{13}C detection of $\omega_1/2\pi$ = 83 kHz. Contact times for the CP step ranged from 5 μs to 5 ms (up to 1 ms for the HETCOR). In directly excited ^{13}C MAS NMR experiments a ^{13}C rf pulse amplitude of $\omega_1/2\pi$ = 60 kHz was applied, and SPINAL-64 heteronuclear decoupling at a rf field amplitude $\omega_1/2\pi$ = 10 kHz was used in high power decoupling experiments (HPDEC) All ^{1}H and ^{13}C spectra were referenced to water at 4.8 ppm and to the tertiary carbon of adamantane at 29.45 ppm,[6] respectively.

Li^+ ion jump rates τ^{-1} were extracted from the temperature dependency of the line width data in Figure 2b through fitting the data to a Boltzmann sigmoid regression curve of the form:

$$\Delta\nu(T) = \left(\frac{\Delta\nu_\infty - \Delta\nu_R}{1 + \exp\left(\frac{T_{point} - T}{a}\right)}\right) + \Delta\nu_R \qquad \text{(S2)}$$

where $\Delta\nu(T)$ is the line width of the central transition at temperature T, $\Delta\nu_\infty$ is the residual line with caused by non-dipolar interactions when motional narrowing is completed, $\Delta\nu_R$ is the line width of the rigid lattice, T_{point} is the temperature of the inflection point and a is a fitting parameter. At the inflection point of the narrowing curve, the mean Li^+ jump rate is expected to be in the order of $2\pi\Delta\nu_R$.

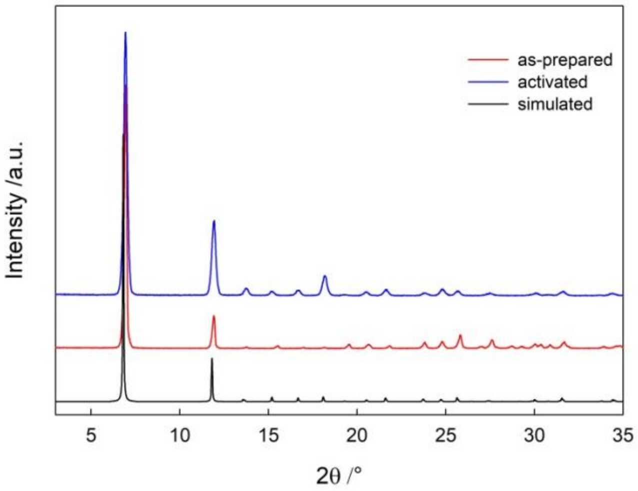

Figure SI-1. XRPD patterns of both pristine (blue) and activated (red) Mg-MOF-74 in comparison with the pattern simulated according to ref [7].

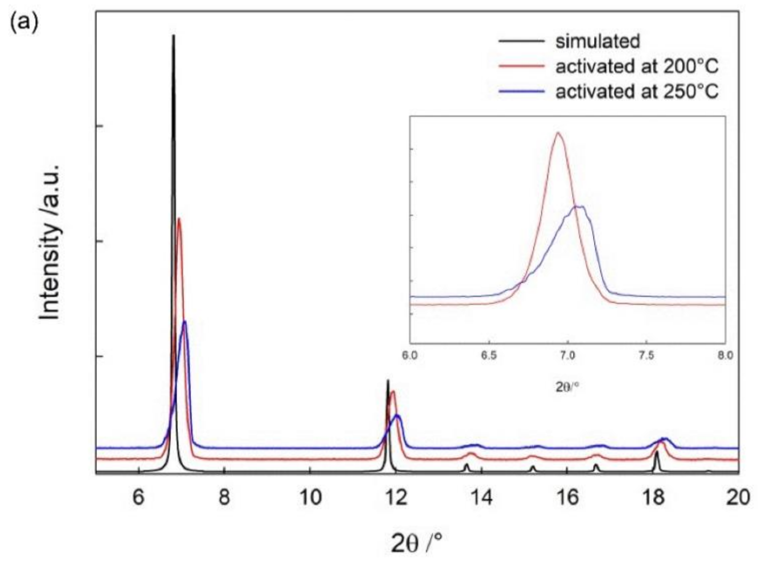

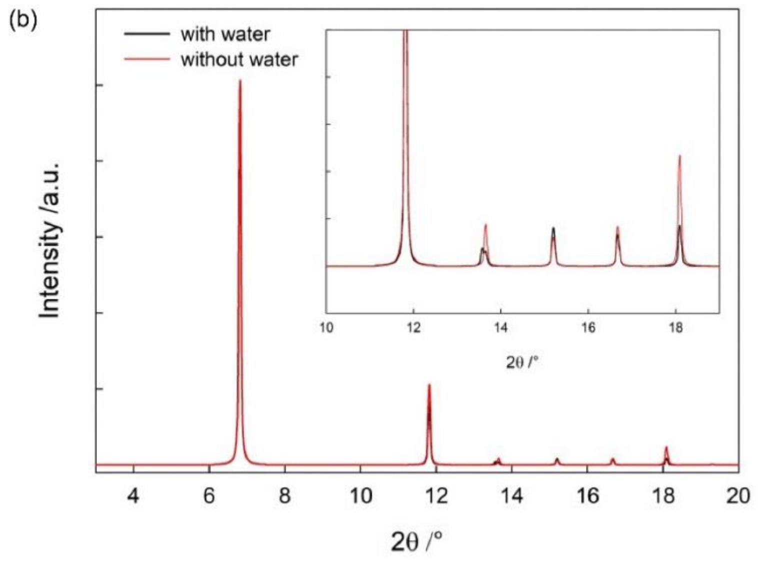

Figure SI-2. (a) Comparison between the calculated pattern of Mg-MOF-74 with and without water [7]; cell parameters and atomic positions are kept fixed in the two models and intensities were normalized at the main peak. (b) Comparison between the XRPD patterns of the Mg-MOF-74 sample activated at two different maximum temperatures, showing the broadening of the peaks as the maximum temperature increases.

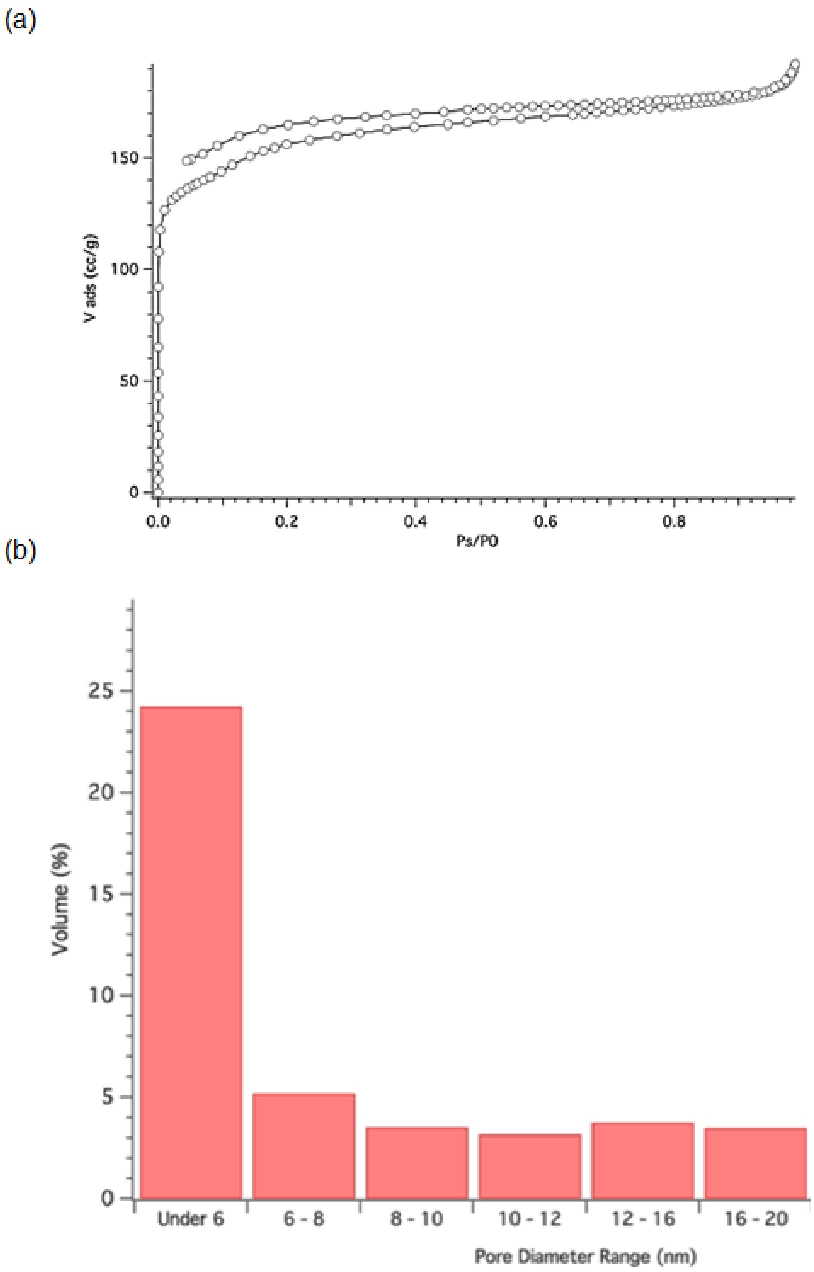

Figure SI-3. (a) N_2 adsorption/desorption isotherms and (b) pore size distribution plot of activated Mg-MOF-74.

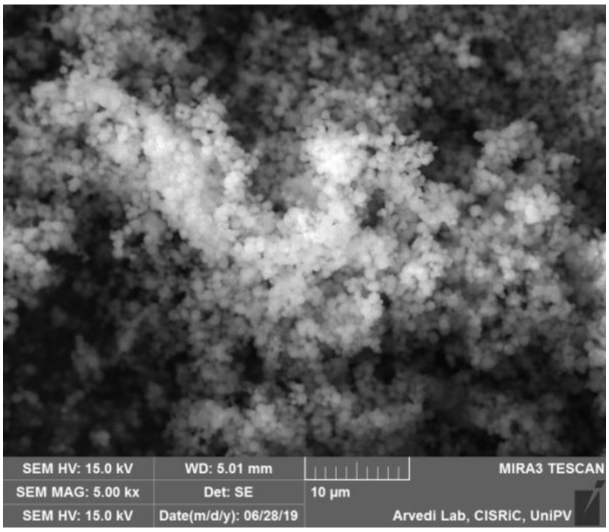

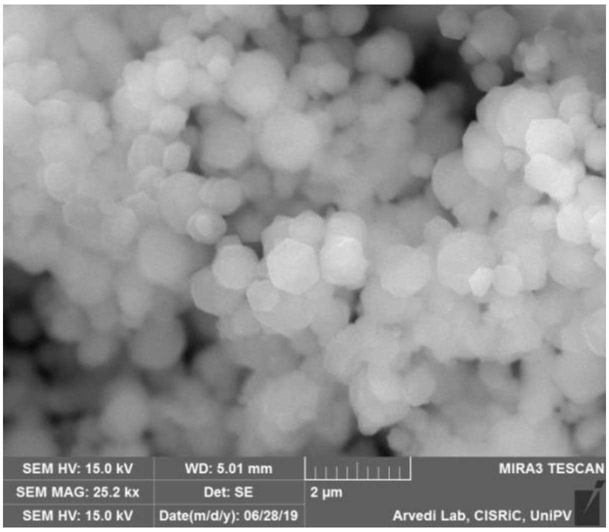

Figure SI-4. SEM images of the as-prepared Mg-MOF-74 sample at two different magnifications.

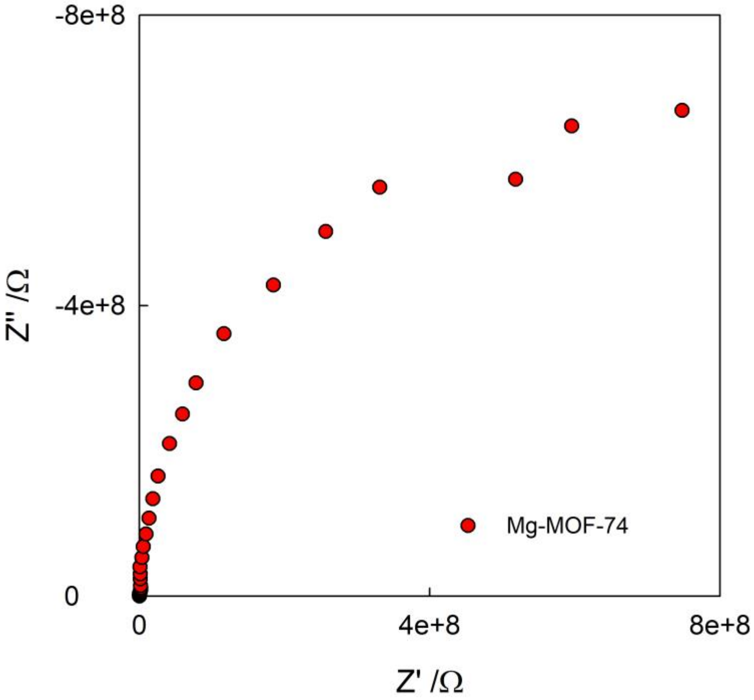

Figure SI-5. Example of Nyquist plot for activated Mg-MOF-74.

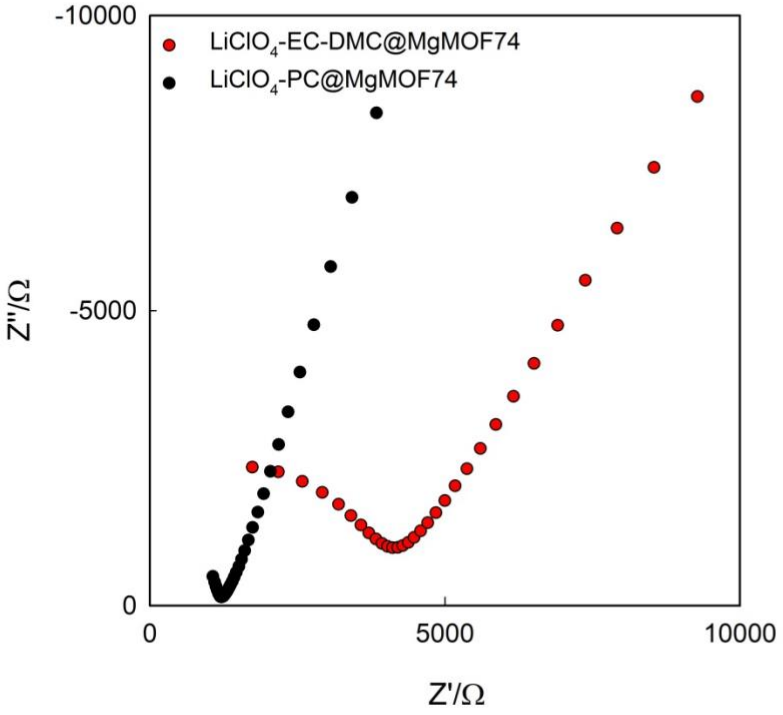

Figure SI-6. Example of Nyquist plots of the investigated MOF-based quasi solid electrolytes collected at room temperature.

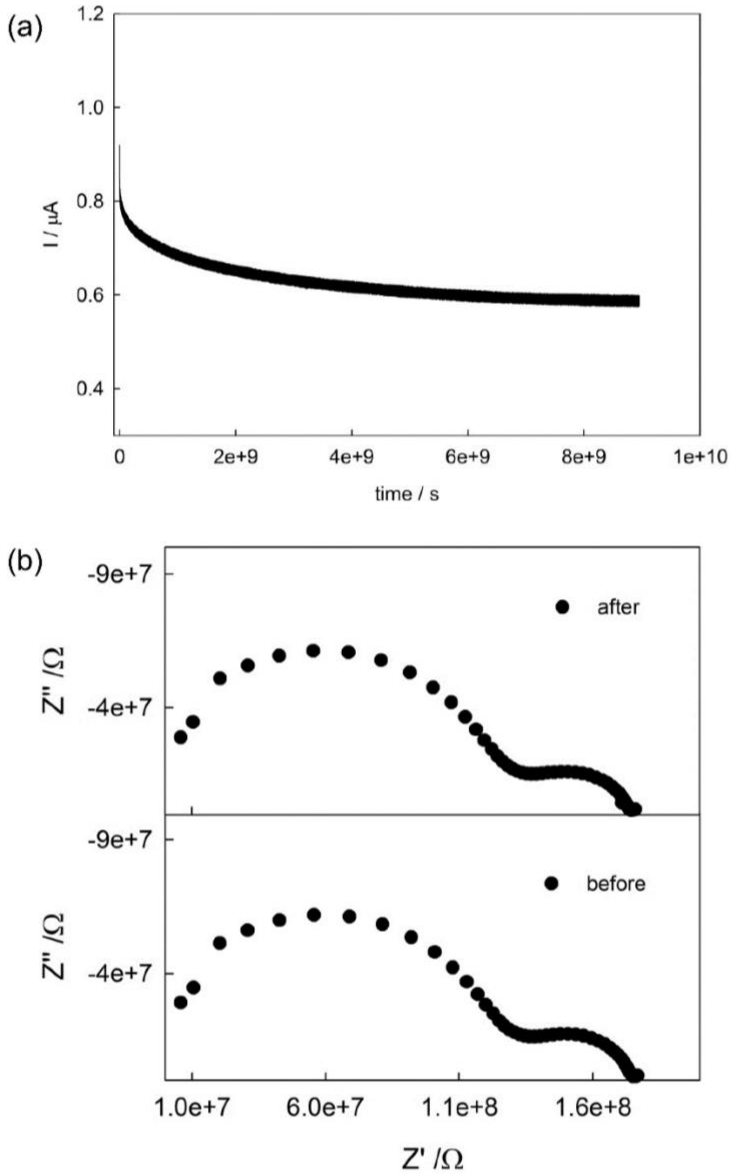

Figure SI-7. (a) Cronoamperometry and (b) impedance spectroscopy for the t^+ determination for $LiClO_4$-EC-DMC@MgMOF74.

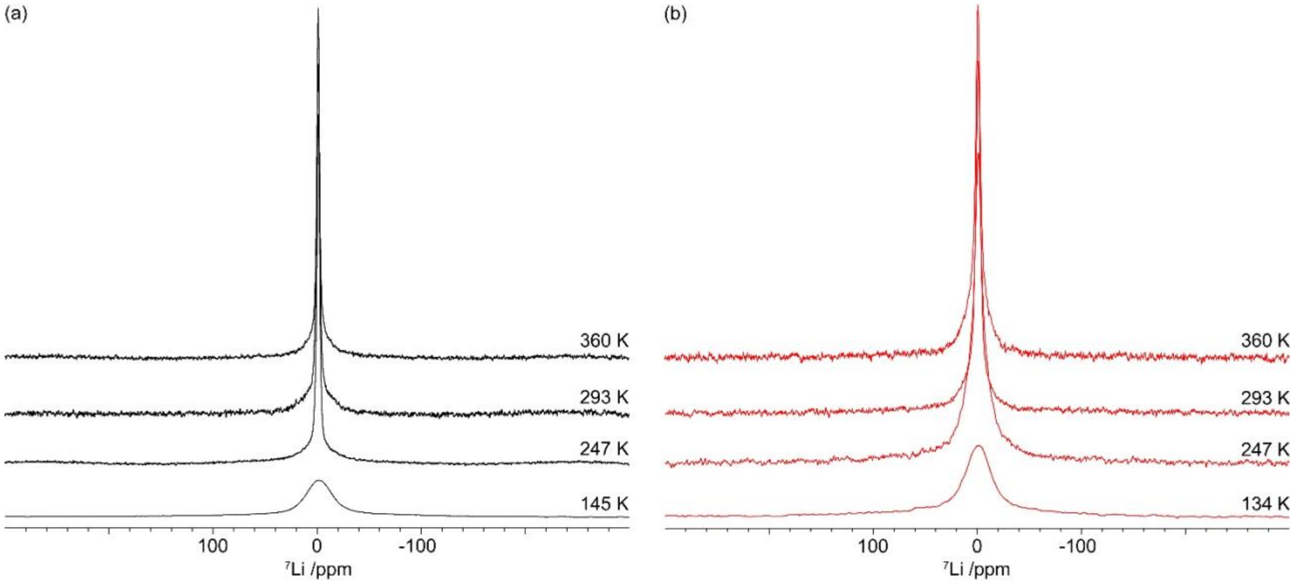

Figure SI-8. ^{7}Li static NMR spectra as a function of temperatures for (a) $LiClO_4$-PC@MgMOF74 (black) and (b) $LiClO_4$-EC-DMC@MgMOF74 (red). The increase in temperature activates the Li^+ ion mobility and results in line width narrowing which occurs at a lower temperature for $LiClO_4$-PC@MgMOF74 than $LiClO_4$-EC-DMC@MgMOF74 indicating faster Li^+ mobility in the former.

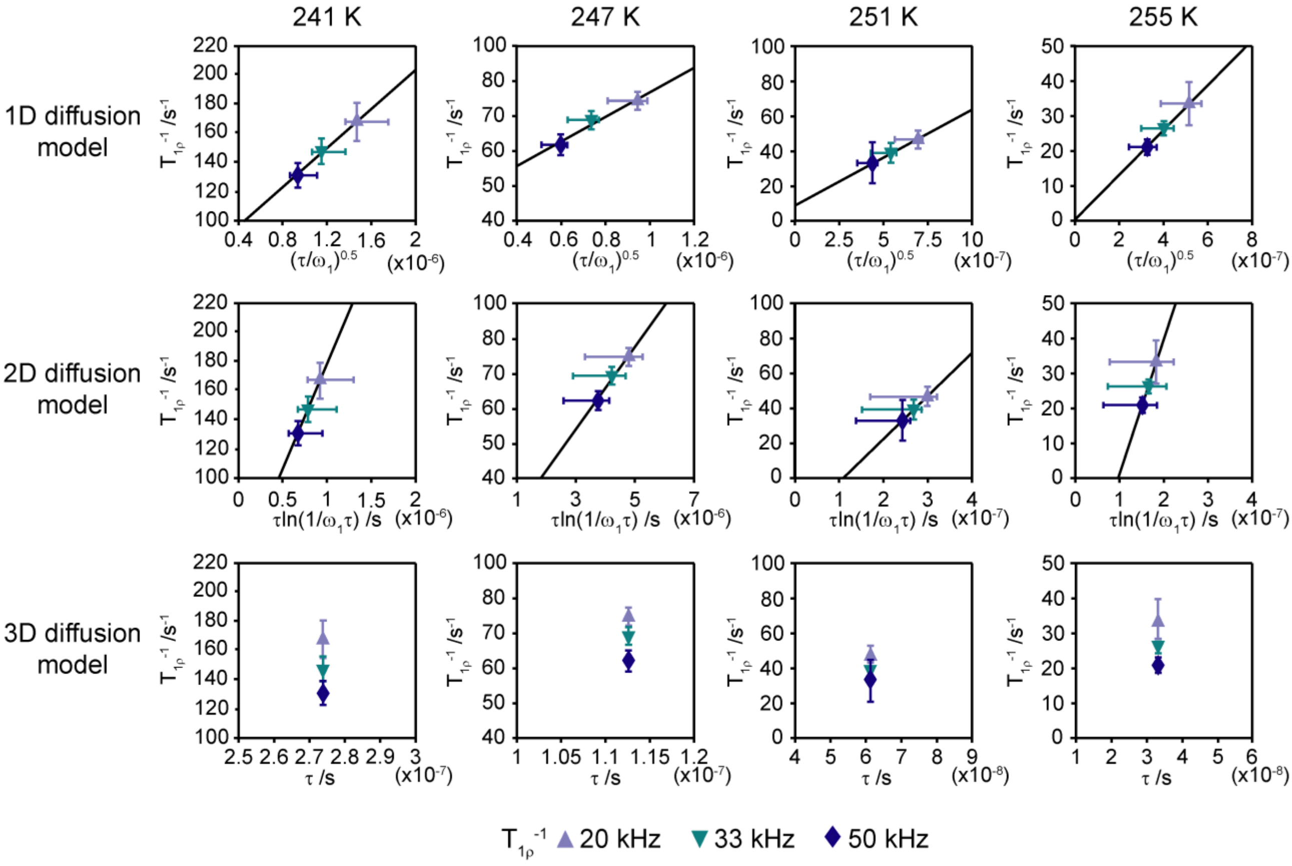

Figure SI-9. Frequency dependence of the NMR SLR $T_{1\rho}^{-1}$ rates of LiClO4-PC@MgMOF74 at 241, 247, 251 and 255 K for one, two and three-dimensional models at spin lock frequencies $\omega_1/2\pi$ of 20 (light purple triangle), 33 (turquois inverted triangle) and 50 kHz (purple diamond) using average Li^+ correlation times τ extracted from $T_{1\rho}^{-1}$ maxima (Figure SI 9). The solid lines correspond to linear fits of $(\tau/\omega_1)^{0.5}$ and $\tau\ln(1/\omega_1\tau)$ for one and two-dimensional diffusion respectively.

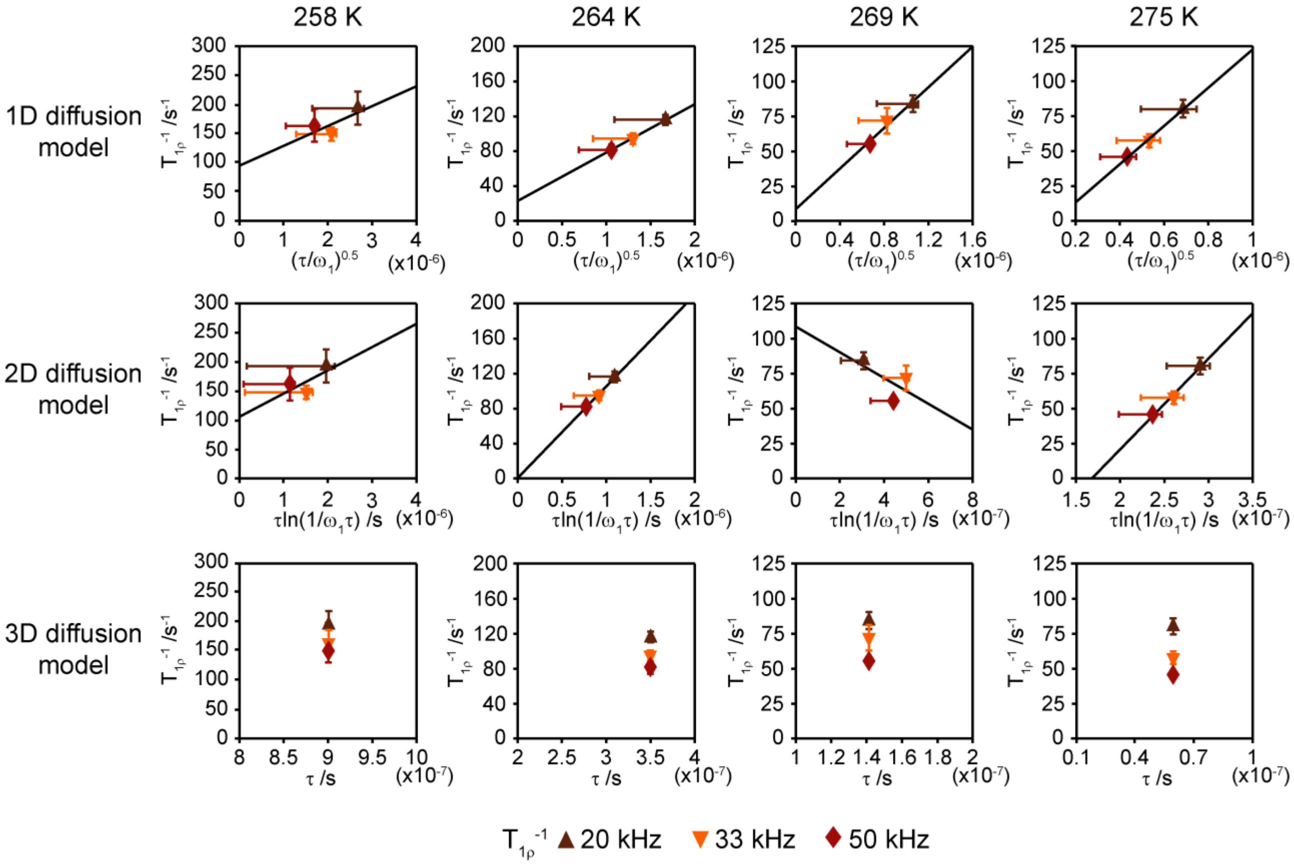

Figure SI-10. Frequency dependence of the NMR SLR $T_{1\rho}^{-1}$ rates of $LiClO_4$-EC-DMC@MgMOF74 at 258, 264, 269 and 275 K for one, two and three-dimensional models at spin lock frequencies $\omega_1/2\pi$ of 20 (brown triangle), 33 (orange inverted triangle) and 50 kHz (red diamond) using average Li^+ correlation times τ extracted from $T_{1\rho}^{-1}$ maxima (Figure SI 9). The solid lines correspond to linear fits of $(\tau/\omega_1)^{0.5}$ and $\tau\ln(1/\omega_1\tau)$ for one and two-dimensional diffusion, respectively.

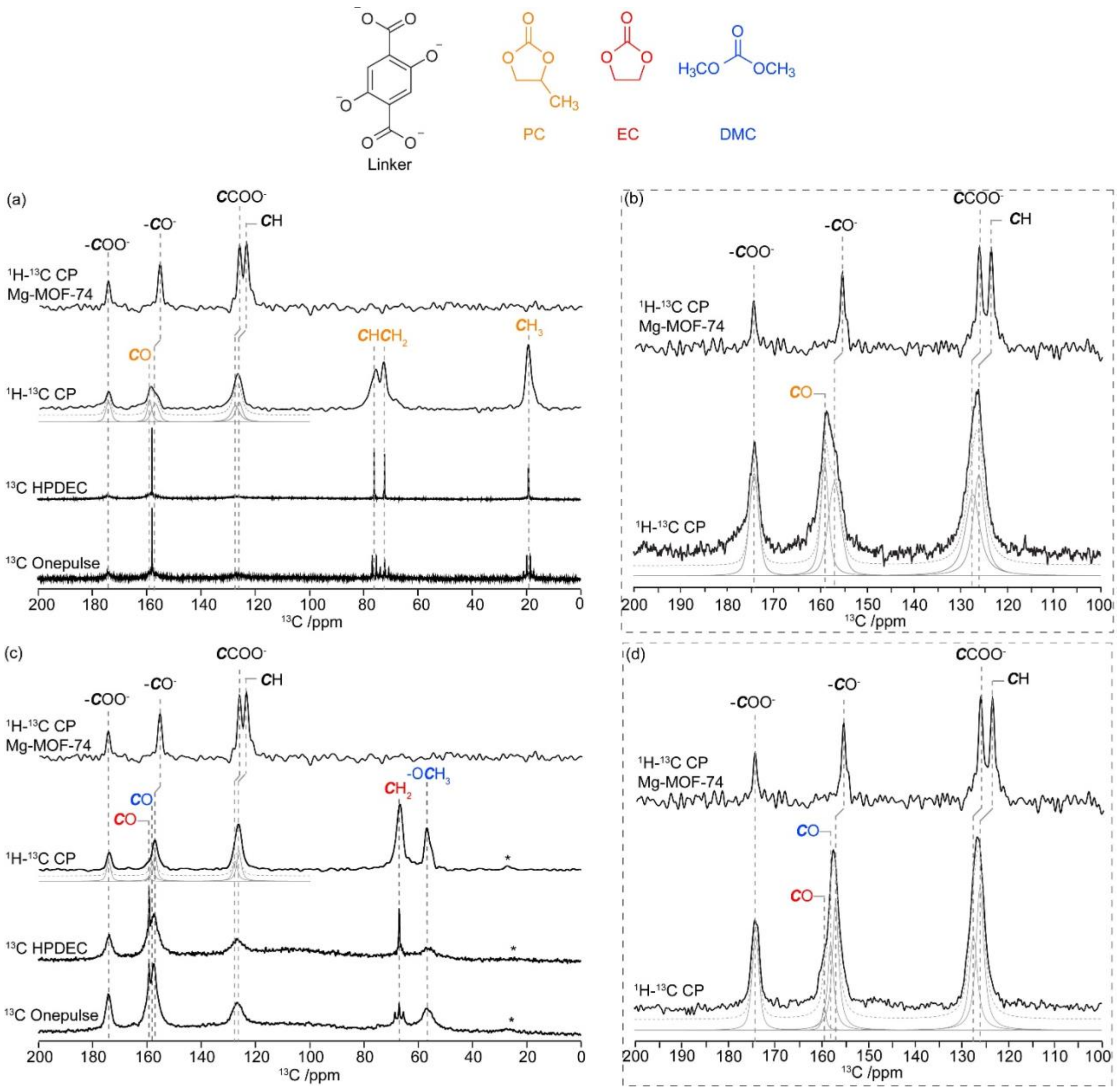

Figure SI-11. Comparison of ^{1}H-1^{3}C CP, high power decoupling ^{13}C (HPDEC) and ^{13}C one pulse NMR spectra for (a,b) $LiClO_4$-PC@MgMOF74 and (c,d) $LiClO_4$-EC-DMC@MgMOF7$_4$. ^{1}H-^{13}C CP Mg-MOF-74 with simulated (dashed grey lines) and deconvoluted spectra (grey lines) are shown on top of each panel. Assignments for the linker, PC, EC, DMC, which chemical structures are shown on top of the figure, are given in black, orange, red, and blue, respectively. Liquid-state species resonances exhibit highly resolved and narrow peaks, and with splitting from scalar J coupling (when applicable) while signals from solid-state are generally broad. The inserts on the right shown magnification of the ^{1}H-^{13}C CP spectra of Mg-MOF-74, ^{1}H-^{13}C CP spectra of $LiCLO_4$-PC@MgMOF74 (insert b) and $LiClO_4$-EC-DMC@MgMOF74 (insert d) in the 200 – 100 ppm region and highlight changes in chemical shifts for ***-C***O-, ***C***COO-, and CH linker signals between pure linker and the quasi-solid samples. Signals labelled with * are spinning sidebands.

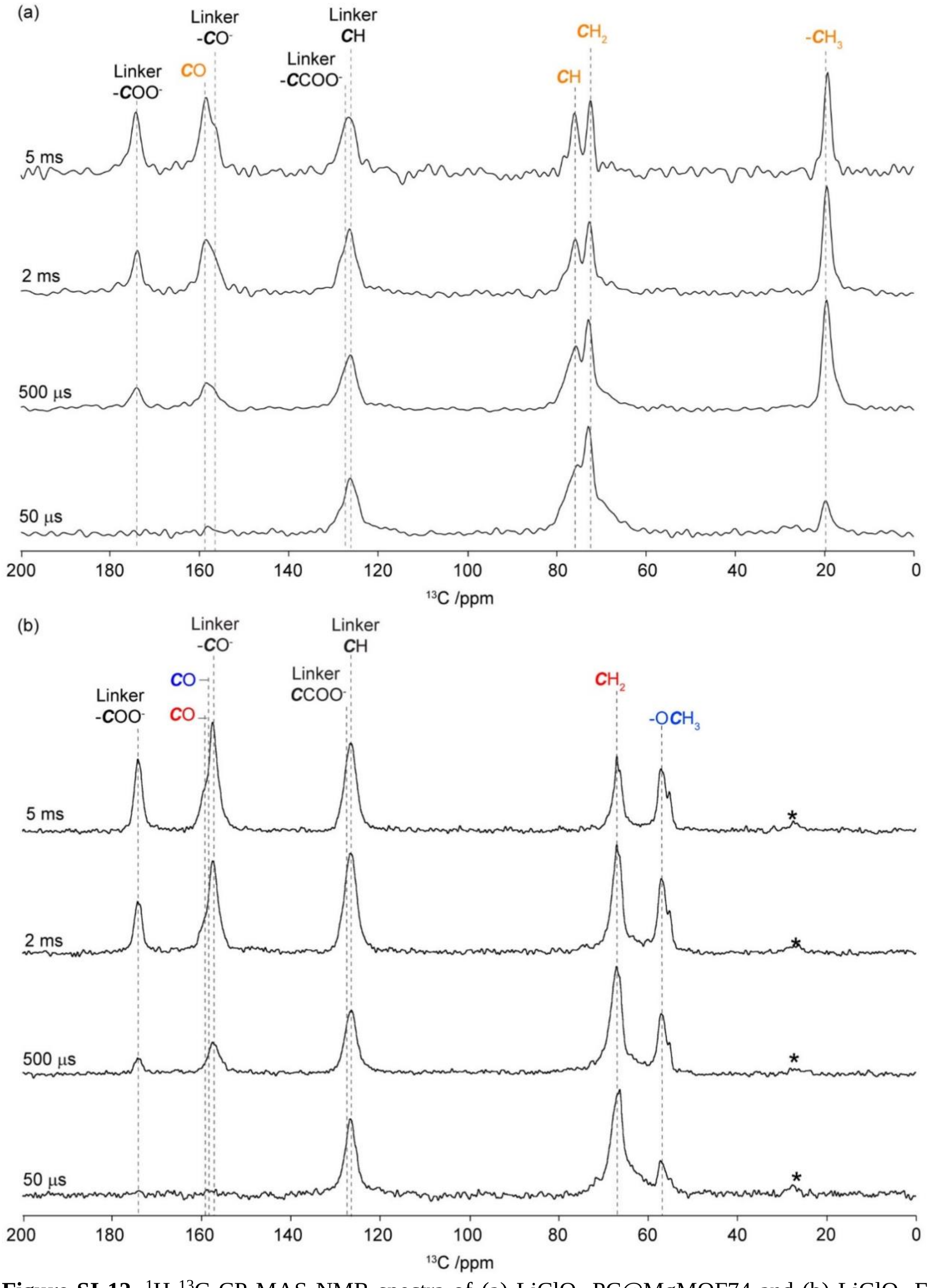

Figure SI-12. ^{1}H-^{13}C CP MAS NMR spectra of (a) $LiClO_4$-PC@MgMOF74 and (b) $LiClO_4$-EC-DMC@MgMOF74 obtained as a function of contact times. The signals of protonated carbons are observed at a very short contact time of 50 μs. Assignments for linker, PC, EC, DMC are given in black, orange, red, and blue, respectively. Signals labelled with * are spinning sidebands.

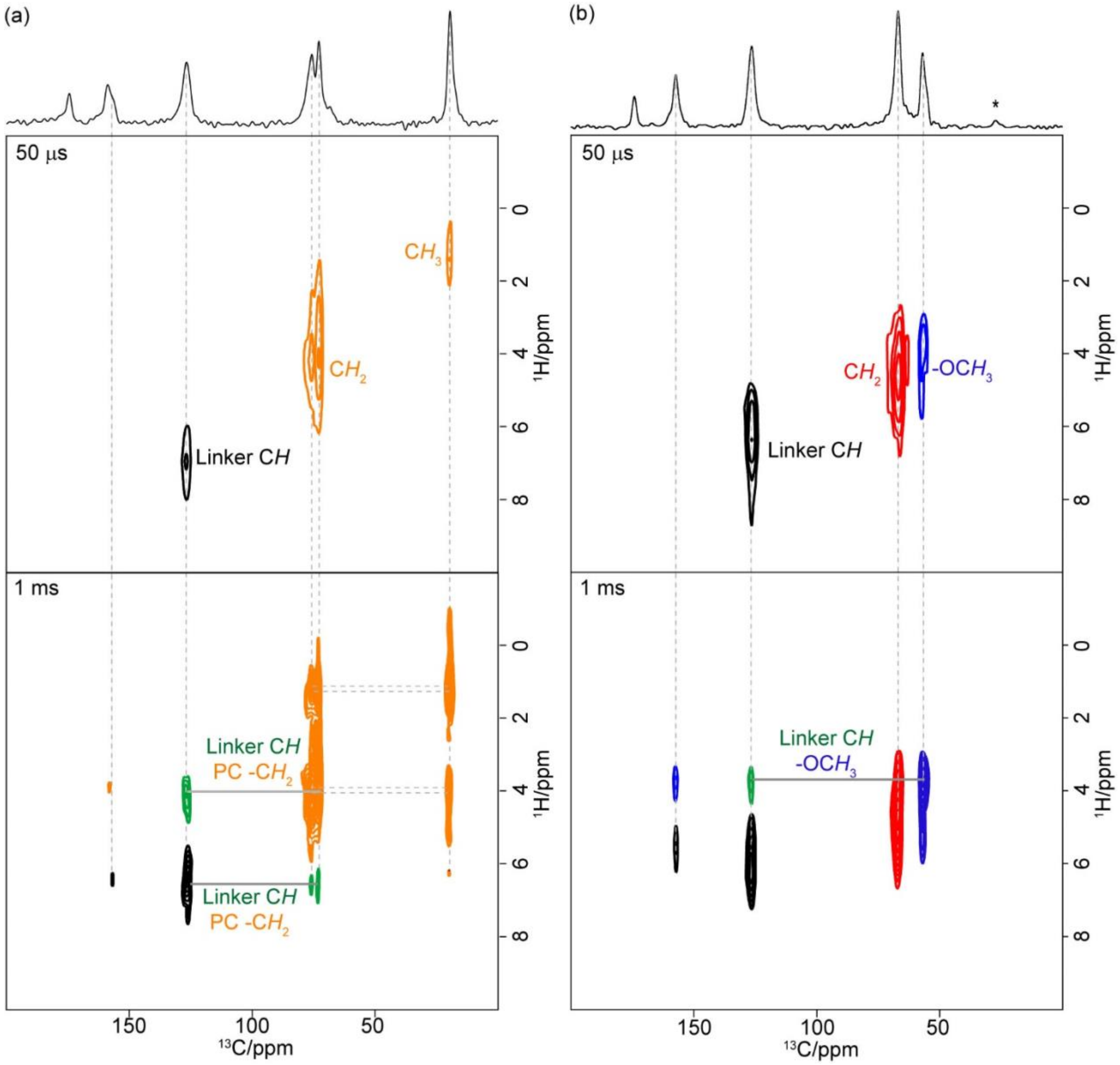

Figure SI-13. 2D ^{1}H-^{13}C HETCOR NMR spectra for (a) $LiClO_4$-PC@MgMOF74 and (b) $LiClO_4$-EC-DMC@MgMOF74 using short and long CP contact times of 50 µs and 1 ms as indicated. The spectra for $LiClO_4$-EC-DMC@MgMOF74 and $LiClO_4$-PC@MgMOF74 show correlations for EC (in red), DMC (blue), PC (orange) and the MOF organic linker (in black). The extra correlations (in green) observed in the spectra obtained with a CP contact time of 1 ms emphasize close proximity between solvent and MOF, suggesting absorption into the pores. On top of each panel are reported the ^{1}H-^{13}C CP MAS NMR spectra of (a) $LiClO_4$-PC@MgMOF74 and (b) $LiClO_4$-EC-DMC@MgMOF74.

Table S1. Room temperature total conductivities and activation energies for some MOF-based hybrid electrolytes.

**Li salt**	**solvent**	**molar concentration**	**σ_{25} (Scm^{-1})**	**E_a (eV)**
**$LiClO_4$**	**EC/DMC**	**1 M**	**$4.6x10^{-5}$**	**0.23**
$LiPF_6$	EC/DMC	1 M	$4.9x10^{-6}$	0.21
LiTFSI	EC/DMC	1 M	$3.7x10^{-5}$	0.27
$LiClO_4$	PC	0.5 M	$4.0x10^{-5}$	0.10
$LiClO_4$	PC	1 M	$9.0x10^{-5}$	0.17
**$LiClO_4$**	**PC**	**2 M**	**$1.4x10^{-4}$**	**0.22**

Table S2. Summary of the computed values obtained from the fits of the line narrowing data (Figure 2b) with equation S2.

Sample	T_{point} (K)	$\Delta\nu_{\infty}$ (kHz)	$\Delta\nu_{r}$ (kHz)	a (a.u)
$LiClO_4$-PC@MgMOF74	216(5)	0.5(1)	4.7(2)	-5.6(7)
$LiClO_4$-EC-DMC@MgMOF74	240(5)	0.85(9)	4.5(2)	-5.4(8)

T_{point}: the temperature of the inflection point, $\Delta\nu_{\infty}$: residual line with caused by non-dipolar interactions when motional narrowing is completed, $\Delta\nu_{r}$ line width of the rigidi lattice, a: fitting parameter.

Table S3. Summary of the activation energies extracted from impedance, ^{7}Li SLR and motional narrowing data.

Sample	$E_{a,\mathrm{ACIS}}$ (eV)	$E_{a,\mathrm{Waugh\text{-}Fedin}}$ (eV)	$E_{a,T1}$ (eV)	$E_{a,T1\rho 3D,\mathrm{LT}}$ (eV)	$E_{a,T1\rho 3D,\mathrm{HT}}$ (eV)	$E_{a,T1\rho 1D,\mathrm{LT}}$ (eV)	$E_{a,JR}$ (eV)
$LiClO_4$-PC@MgMOF74	0.22(1)	~0.3	0.19(1)	0.23(9)	0.64(7)	0.18(4)	0.79(14)
$LiClO_4$-EC-DMC@MgMOF74	0.23(3)	~0.4	0.35(6)	0.40(9)	0.49(8)	0.18(1)	0.93(8)

LT: low temperature, HT: high temperature, JR: jump rate (Figure 4)

Table S4. ^{13}C NMR spectral assignments chemical shifts in ppm for all the sample used

Sample	Linker ***C***OO$^-$	EC -***C***=O	PC -***C***=O	DMC ***C***=O	Linker -***C***O$^-$	Linker -***C***-COO$^-$	Linker -***C***H	PC -***C***H	PC -***C***H_2	EC -***C***H_2-	DMC -O***C***H_3	PC -***C***H_3
Mg-MOF-74	174.6	n.a.	n.a.	n.a.	155.5	126.3	123.8	n.a.	n.a.	n.a.	n.a.	n.a.
$LiClO_4$-PC@MgMOF74	174.0	n.a.	158.0	n.a.	157.4	127.7	126.3	75.9	72.3	n.a.	n.a.	19.2
$LiClO_4$-EC-DMC@MgMOF74	174.0	159.2	n.a.	158.3	157.4	127.7	126.3	n.a.	n.a.	67.0	65.1	n.a.

n.a.:not applicable

References

[1] L. Garzón-Tovar, A. Carné-Sánchez, C. Carbonell, I. Imaz, D. Maspoch. Optimised room temperature, water-based synthesis of CPO-27-M metal–organic frameworks with high space-time yields. *J. Mater. Chem. A* **2015**, *3*, 20819–20826.

[2] J. Evans, C. A. Vincent, P. G. Bruce. Electrochemical measurement of transference numbers in polymer electrolytes *Polymer* **1987**, *28*, 2324–2328.

[3] P. A. Beckmann, C. Dybowski. A Thermometer for Nonspinning Solid-State NMR Spectroscopy. *J. Magn. Reson.* **2000**, *146*, 379–380.

[4] A. Bielecki, D. P. Burum. Temperature Dependence of ^{207}Pb MAS Spectra of Solid Lead Nitrate. An Accurate, Sensitive Thermometer for Variable-Temperature MAS. *J. Magn. Reson., Series A* **1995**, *116*, 215–220.

[5] B. M. Fung, A. K. Khitrin, K. Ermolaev. An Improved Broadband Decoupling Sequence for Liquid Crystals and Solids. *J. Magn. Reson.* **2000**, *142*, 97–101.

[6] C. R. Morcombe, K. W. Zilm. Chemical shift referencing in MAS solid state NMR. *J. Magn. Reson.* **2003**, *162*, 479–486.

[7] S. E. Henkelis, S. M. Vornholt, D. B. Cordes, A. M. Z. Slawin, P. S. Wheatley, R. E. Morris. A single crystal study of CPO-27 and UTSA-74 for nitric oxide storage and release. *CrystEngComm.* **2019**, *21*, 1857–1861.
